# Adjustable prosthetic sockets: a systematic review of industrial and research design characteristics and their justifications

**DOI:** 10.1186/s12984-023-01270-0

**Published:** 2023-11-06

**Authors:** Michael Baldock, Nicolaas Pickard, Michael Prince, Sarah Kirkwood, Alix Chadwell, David Howard, Alex Dickinson, Laurence Kenney, Niamh Gill, Sam Curtin

**Affiliations:** 1 School of Health and Society at the University of Salford, Salford, UK; 2 School of Engineering at Newcastle University, Newcastle upon Tyne, UK; 3 School of Engineering at the University of Southampton, Southampton, UK

**Keywords:** Prosthetic, Artificial limb, Prosthesis design, Prosthetic socket, Socket, Residual limb, Prosthetic interface, Adjustable socket, Adjustable-volume, Systematic review

## Abstract

**Background:**

The prosthetic socket is a key component that influences prosthesis satisfaction, with a poorly fitting prosthetic socket linked to prosthesis abandonment and reduced community participation. This paper reviews adjustable socket designs, as they have the potential to improve prosthetic fit and comfort through accommodating residual limb volume fluctuations and alleviating undue socket pressure.

**Methods:**

Systematic literature and patent searches were conducted across multiple databases to identify articles and patents that discussed adjustable prosthetic sockets. The patents were used to find companies, organisations, and institutions who currently sell adjustable sockets or who are developing devices.

**Results:**

50 literature articles and 63 patents were identified for inclusion, representing 35 different designs used in literature and 16 commercially available products. Adjustable sockets are becoming more prevalent with 73% of publications (literature, patents, and news) occurring within the last ten years. Two key design characteristics were identified: principle of adjustability (inflatable bladders, moveable panels, circumferential adjustment, variable length), and surface form (conformable, rigid multi-DOF, and rigid single DOF). Inflatable bladders contributed to 40% of literature used designs with only one identified commercially available design (n = 16) using this approach. Whereas circumferential adjustment designs covered 75% of identified industry designs compared to only 36% of literature devices. Clinical studies were generally small in size and only 17.6% of them assessed a commercially available socket.

**Discussion:**

There are clear differences in the design focus taken by industry and researchers, with justification for choice of design and range of adjustment often being unclear. Whilst comfort is often reported as improved with an adjustable socket, the rationale behind this is not often discussed, and small study sizes reduce the outcome viability. Many adjustable sockets lack appropriate safety features to limit over or under tightening, which may present a risk of tissue damage or provide inadequate coupling, affecting function and satisfaction. Furthermore, the relationship between design and comfort or function are rarely investigated and remain a significant gap in the literature. Finally, this review highlights the need for improved collaboration between academia and industry, with a strong disconnect observed between commercial devices and published research studies.

**Supplementary Information:**

The online version contains supplementary material available at 10.1186/s12984-023-01270-0.

## Introduction

The human-prosthesis interface, most commonly a personalised ‘socket’, is often identified as one of the most essential components of a prosthesis [1]. In conventional modern socket design, the geometry of the individual’s residual limb is captured using plaster casts or 3D scanning, following which rectifications are made to the shape to load or unload specific areas of the individual’s residual limb. Once made these sockets do not change in shape. The design and manufacture of a satisfactory fixed geometry prosthetic socket (hereafter ‘fixed geometry sockets’), particularly for primary patients, can involve several iterations over weeks or months. Fixed geometry sockets are also often difficult to implement when working in low resource settings, due to a lack of sophisticated and accessible prosthetic facilities, difficulty in obtaining materials (such as plaster or polypropylene), and reduced access to the clinical expertise required to implement the iterative fitting process [2].

Despite being personalised, prosthetic sockets are often identified as the most problematic prosthetic component for users [3, 4]. Residual limb volume fluctuations, which can be caused by diet and activity level [5], comorbidities such as diabetes, or dialysis, or more long term residual limb changes such as muscle atrophy and growth in a child, mean that the fit of fixed geometry sockets will inevitably alter over time [3]. Residual limb volume fluctuations are larger for individuals with lower-limb amputations. Transtibial residua can reduce in volume by up to 35% following amputation due to post-operative oedema [6] and change by up to 6.5% during short periods of activity [7]. Poor fit can lead to issues regarding prosthetic function, with poor mechanical coupling (the change in socket pose relative to the limb, under a given load. i.e. a stiffness or compliance measure) making it difficult for the person to use their prosthesis in a precise and confident manner. Poorly fitting sockets often lead to discomfort due to skin breakdown and tissue damage, which can have major long-term consequences. Skin damage has been found to occur in 36% to 63% of lower limb prosthesis users [8–10], with similar occurrence levels found in upper limb users [11], however the incidence and severity is less well documented for people using upper limb devices. These factors are often cited as leading causes of prosthetic abandonment, particularly for people with upper limb absence, who often favour their intact limb over their prosthesis when comfort and function are compromised [12]. Prosthetic abandonment often results in activity reduction which can lead to further health complications as well as a reduction in social participation and an increase in psychosocial issues [12].

In an effort to address issues experienced with fixed geometry prosthetic sockets, adjustable sockets have been considered as an alternative since the medieval times [13]. The increasing popularity of adjustable sockets is resulting in an influx of designs being produced by both commercial and research groups, with a wide range of concepts using different approaches to both how the socket shape is varied, and how that is controlled. Off-the-shelf devices are also being developed to a lesser extent, and there is specific interest into how these can be utilised within low resource settings, to reduce the time, clinical expertise and materials which are required to fit and manufacture conventional sockets [4]. Previous reviews of adjustable sockets have categorised them by their method of manufacture: off-the-shelf, where a prosthesis is purchased pre-made to a set size with small adjustments made to suit the patient; modular, where a kit of parts is delivered to and assembled by the clinician with modifications to parts made to suit the patient; and custom, where the prosthesis is made by the clinician or manufacturer to match the patients residuum shape with no limitation on shape due to pre-made components [14, 15]. This categorisation is useful for fabrication but provides no insight into how or where the shape of the socket changes when adjusted, which are significant due to how they influence the stability, comfort and tissue health dimensions listed above.

It is worth noting that approaches to mitigate, rather than accommodate, limb volume fluctuations are available. An example is elevated vacuum sockets which have been shown in lower-limb studies to reduce the rate of fluid loss during use [16] and improve limb oxygenation [17].However, these systems cannot be used by all individuals, such as those with skin conditions, short residua, or a bulbus distal end.

This paper reports a systematic review of the design of adjustable sockets, both within literature and from commercial sources. The majority of commercially available adjustable sockets are not mentioned in literature, so the review’s scope was expanded to include patents. The objective is to cluster designs based on shared characteristics to provide a classification which describes how the socket adjustability functions, to compare how the motivation behind designs varies between research and commercially available devices, compare how these change depending on the target amputation level for each design, and to comment on the clinical findings of each design.

## Search methodology

This review focusses on the design of prosthetic sockets which are adjusted manually by the user or clinician, or automatically by the device, with irreversible adaptations, such as cutting components to size, not being classed as adjustable. The search string developed by Nylander et al. [18] to identify literature relevant to prosthetics was used as the basis for both the literature and industry searches. The aspects of their search criteria that concentrated on the prosthesis and its design were combined with our additional search terms to define adjustability (Table 1). Article and patent relevance was determined using the PRISMA framework [19] consisting of the following four criteria: (1) they must discuss a prosthetic socket, (2) the socket must be intended to replace appendicular skeletal limbs, and (3) the socket must be adjustable in a controllable manner. Finally, (4) articles were excluded if they described a flexible socket which changes shape due to specific stiffness characteristics, described an adjustable component intended to comprise part of an alternative prosthesis component such as the liner, focused upon control loop or actuator design, or lacked sufficient design information in the report to classify the device. This screening process was conducted by two authors, with a third used for instances where a consensus was not reached.

**Table 1:** Search criteria and databases

Material	Search criteria	Databases
Literature	*(Prosthetic OR Prosthesis OR Prostheses OR Prosthetics OR “Prosthesis Design” OR “Artificial Limb”) AND (Socket) AND (Adjustable OR Adaptable OR Adaptive OR Variable OR Active OR Adjustments OR”Adjustable-Volume”) NOT (Dental OR tooth OR teeth OR orbital OR oral OR Arthroplasty OR Hip OR Implant OR Wear OR Acetabular)* + *Filter for EN or FR Language*	Web of Science—Topic
		Google Scholar—Titles
		IEEE—All Metadata
		PubMed—Title and Abstract
		ProQuest Central—NOFT (Everywhere except full text)
		EBSCOhost—Title and Abstract
Industry	*((ta* = *"Prosthetic" OR ta* = *"Prosthesis" OR ta* = *"Prostheses" OR ta* = *"Prosthetics" OR ta* = *"Prosthesis Design" OR ta* = *"Artificial Limb") AND (ta* = *"Socket" OR ta* = *"System") AND (ta* = *"Adjustable" OR ta* = *"Adaptable" OR ta* = *"Adaptive" OR ta* = *"Variable" OR ta* = *"Active" OR ta* = *"Adjustments" OR ta* = *"Adjustable-Volume")) NOT (ta* = *"Dental" OR ta* = *"tooth" OR ta* = *"teeth" OR ta* = *"orbital" OR ta* = *"oral" OR ta* = *"Arthroplasty" OR ta* = *"Hip" OR ta* = *"Implant" OR ta* = *"Wear" OR ta* = *"Acetabular")* + *Filter for EN Language & A61F2 IPC Main group*	Google Patents—Title
		Google Scholar Patents
		Espacenet—Title and Abstract
		ProQuest Central (News)—NOFT

For the literature aspect of this review, the online databases Web of Science, Google Scholar, IEEE, PubMed, ProQuest Central and EBSCOhost were searched in June 2022 using the search criteria detailed in Table 1. Further to this, the reference list of each article was consulted to identify additional articles not found in the initial search. From this, duplicates were removed, and the titles were assessed to remove any articles which did not discuss prosthetic sockets. Finally, the abstracts (and when unclear the full text) were read to determine relevance. The search was validated by checking the results for previously known articles on this topic.

For the industrial design aspect of this review, databases Google Patents and Espacenet were searched in September 2022 alongside ProQuest News using the search criteria detailed in Table 1. The results were subjected to the same exclusion criteria as the literature search. As patents and news articles lack an abstract, after screening by title the patents were screened using their front pages whereas the news articles were read in full if their relevance was unclear after reading the title. From the resulting patents and news articles, commercial socket designs and associated companies were linked where possible, with further known designs or companies not identified by the search added manually. In June 2023 both searches were repeated to capture any newly published material. The set of adjustable sockets identified in the two searches was analysed, from the descriptions and images available, by how they are adjusted and how adjusting them influences the contact between the residuum and socket. Designs with similar features were grouped to assess whether higher level trends were present.

## Results

**Fig. 1:**
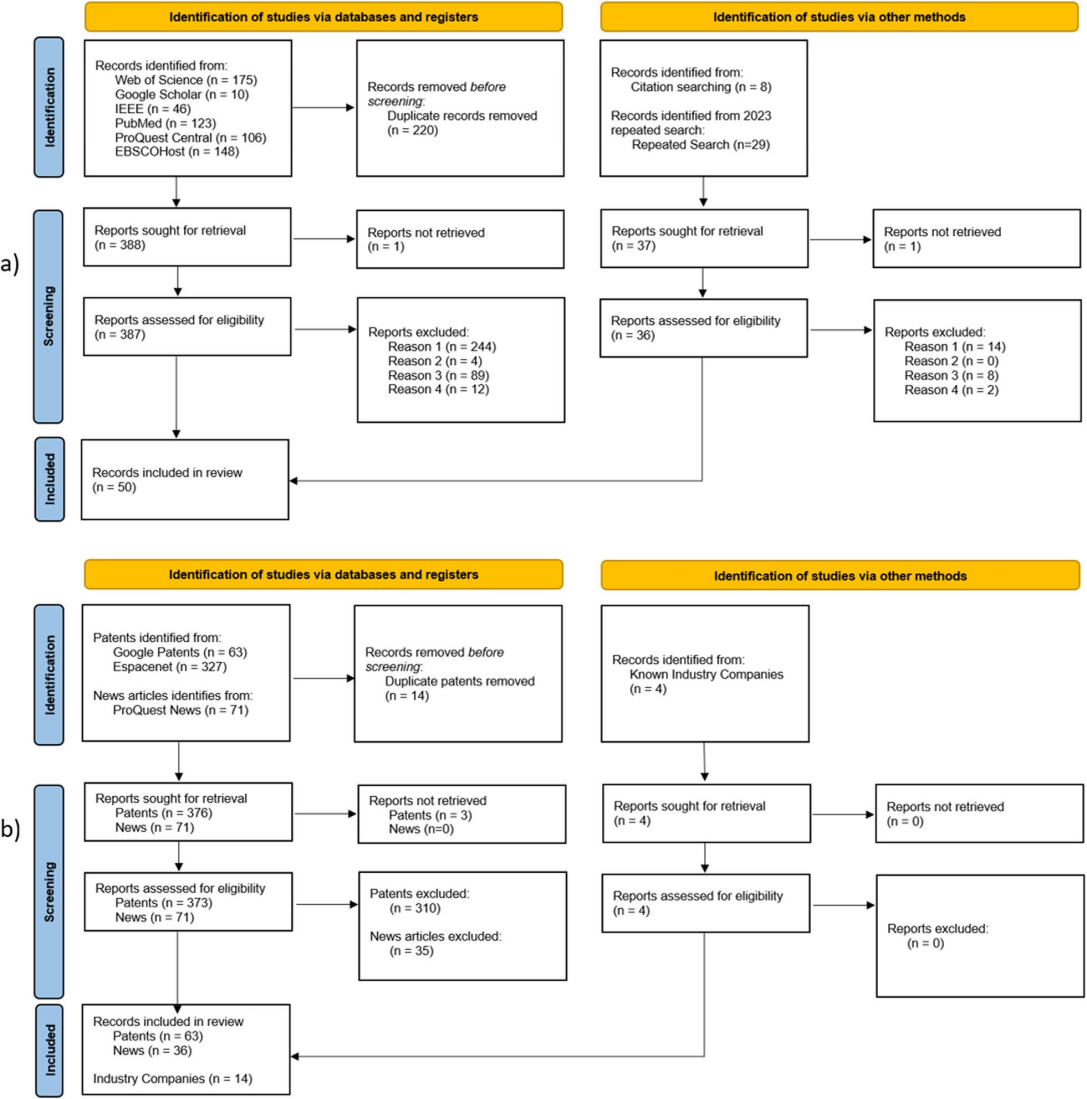
PRISMA [19] flow diagrams detailing the systematic search processes; **a** literature search, **b** industry search

The literature search identified 38 articles, with seven additional articles added through snowballing, and 5 added from the repeated search in 2023 (Fig. 1). The 50 articles spanned a total of 35 different socket designs. The patent search identified 63 patents of which 22 were linked to ten companies with active products and four university institutions (Additional file 1: Appendix—Table S1). The news articles linked to eight active companies and one research institution, all of which had been identified via the patent search. Finally, four additional companies known to the authors were added [20–23], giving 14 companies that currently provide adjustable sockets. Seven of the 14 companies produce prostheses for multiple levels of amputation. If multiple products within a company shared the same design characteristics, then these were grouped as one design. This analysis led to the identification of 16 commercially available designs (Additional file 1: Appendix—Table S2).

**Fig. 2:**
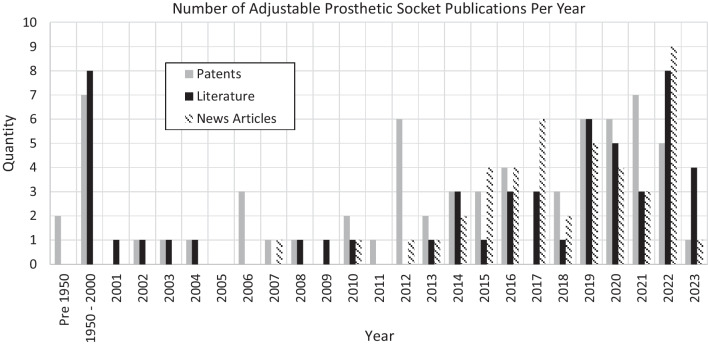
Number of patents, literature papers, and news articles published each year from the search results

The patent search identified 73% of the industrial designs discussed in this review. One identified patent was connected to Washington University [24], and this team also contributed to 22% of the literature articles, showing that they are a key player in the field. The authors from the three other universities who released patents are not linked to any identified literature results. Twelve of the identified patents were linked to companies that did not provide a currently available adjustable socket (Additional file 1: Appendix—Table S1). From the date of the patents, it can be assumed that some come from companies which no longer exist, and others describe designs which are either no longer available or yet to come to market. 73% of identified patents and literature publications fell within the last ten years (Fig. 2).

### Results—adjustable socket categorisation

Based on the descriptions and images available for each design in the report or webpage available, two key design characteristics affect how the socket geometry changes shape. These are the principle of adjustability and the form of the surface that contacts with the residuum when adjusted (Table 2). These two factors were used to group the designs from our search results (Additional file 1: Appendix—Tables S2 and S3). One commercially available product [25] is provided as a system rather than a manufactured socket. They recommend different designs which places it in multiple principle of adjustability categories. In this case, as the surface form can only be evaluated once manufactured, it is excluded from surface form categorisation under industry designs.

**Table 2:** Definitions of design characteristic groups extracted from results

A) Principle of adjustability		Brief description	Design distribution		
			Literature		Industry designs (N = 16)
			Articles (N = 50)	Designs (N = 35)	
Inflatable bladders		A rigid socket containing one or more fluid/air-filled bladders, whose pressure can be adjusted to alter their volume and consequently the internal socket geometry	17 (34.0%)	14 (40.0%)	1 (6.3%)
Moveable panels	Floating	A rigid socket containing one or more adjustable panels whose position translate radially when adjusted	14 (28.0%)	8 (22.9%)	4 (25.0%)
	Hinged	A rigid socket containing one or more adjustable panels whose positions rotate into contact with the residuum when adjusted	3 (6.0%)	3 (8.6%)	1 (6.3%)
Circumferential adjustment	Gap/Overlap	Sockets which can be adjusted by altering their internal circumference by the socket walls either moving or flexing, either at a specific height or throughout the whole height of the socket	9 (18.0%)	7 (20.0%)	8 (50.0%)
	Struts		9 (18.0%)	4 (11.4%)	4 (25.0%)
Variable length		A socket whose internal volume can be varied by changing its length	4 (8.0%)	4 (11.4%)	4 (25.0%)

B) Surface form		Brief description	N = 50	N = 35	N = 15
Conformable		A soft surface profile whose shape deforms to match the residuum upon contact	18 (36.0%)	16 (45.7%)	6 (40.0%)
Rigid	Multi-DOF	A rigid surface which can accommodate small movements in one or more degrees of freedom (DOF) upon contact with the residuum	8 (16.0%)	5 (14.3%)	3 (20.0%)
	Single DOF	A rigid surface which is fixed in all DOF except for its predefined movement governed by the control mechanism in the adjustable socket	24 (48.0%)	14 (40.0%)	6 (40.0%)

#### Principle of adjustability

The most common adjustment method used in literature was inflatable bladders, whereas this was the least popular approach amongst commercially available designs. Moveable panels showed similar distributions across literature and industry, with hinged designs being the least popular. Only 14% of literature designs included multiple principles of adjustability, compared to 25% in industry. In four of these designs, variable length was a secondary principle of adjustment alongside circumferential adjustment, and one design detailing a concept containing moveable panel and circumferential adjustment. Most adjustability mechanisms can be user-adjusted, however in some designs length adjustment can only be implemented by the clinician.

From the 34 studies identified, the median number of participants is ten, however eight studies only included a single participant. Some of the case studies were focused on individuals with specific needs such as a bulbous residuum [26], whilst most others were aimed at demonstrating a proof of concept of a new design.

##### Inflatable bladders

**Fig. 3:**
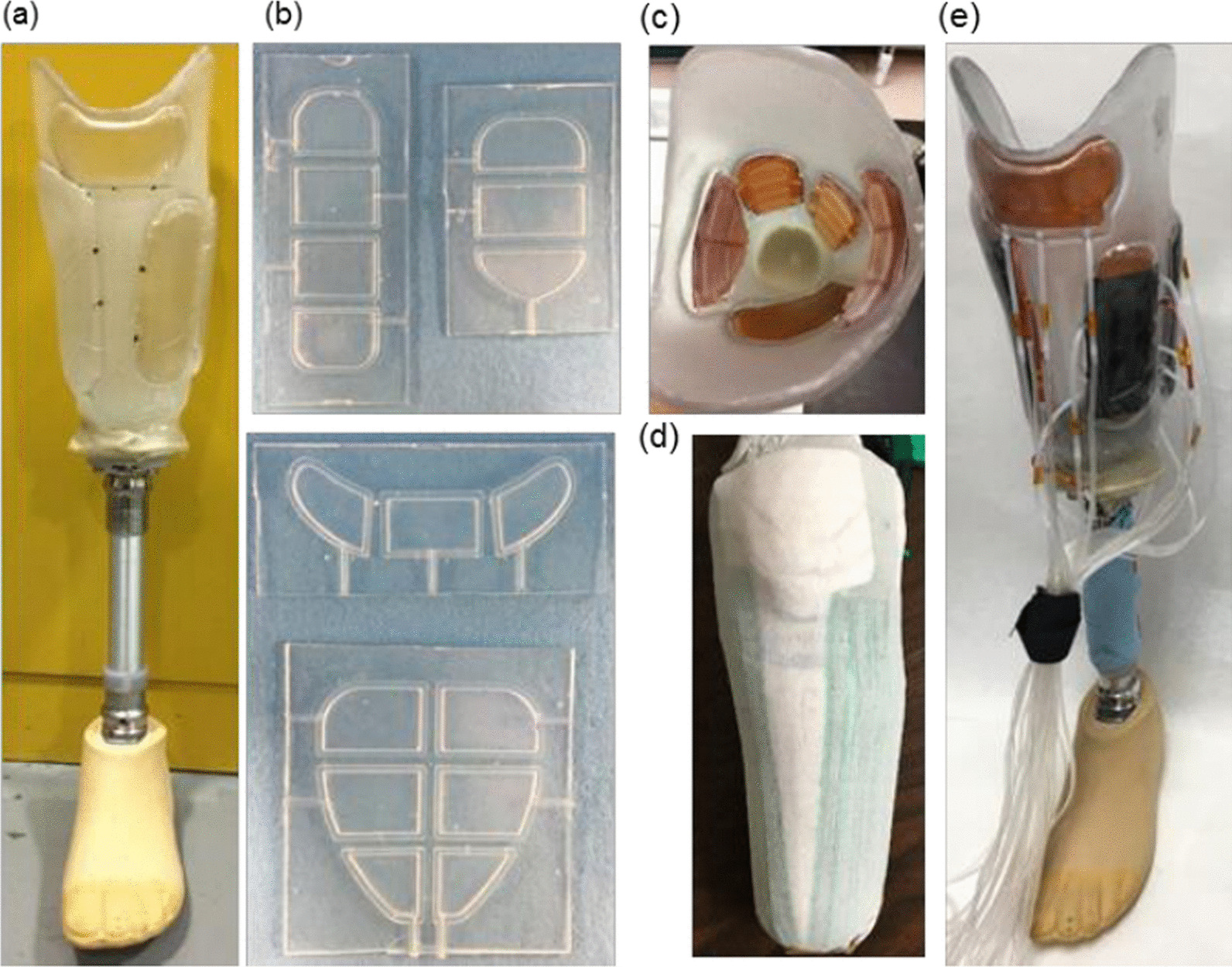
Example of a prosthetic socket including the inflatable bladder principle of adjustability by Carrigan et al. [78]

Inflatable bladders could be a part of the liner (e.g. Sanders et al. [27]), or socket (e.g. Seo et al. [28]), both with similar purposes and outcomes. However, here we are only discussing designs intended to be incorporated into the socket. From the 17 research articles on inflatable bladders, only seven reported undertaking studies involving participants, three of which included only one participant. All the inflatable bladder designs, both commercial and research, were classified as conformable surface form (Fig. 3).

The sole industry design by Epoch Medical only contains one bladder on the posterior surface of a transtibial socket. The associated patent and product has a pump located at the socket’s distal end, before the pylon, which automatically adjusts the bladder pressure [29]. However a more recent patent and associated website details a new manually adjustable bladder system where the pump is located on the posterior aspect of the socket [30, 31].

##### Moveable panels

**Fig. 4:**
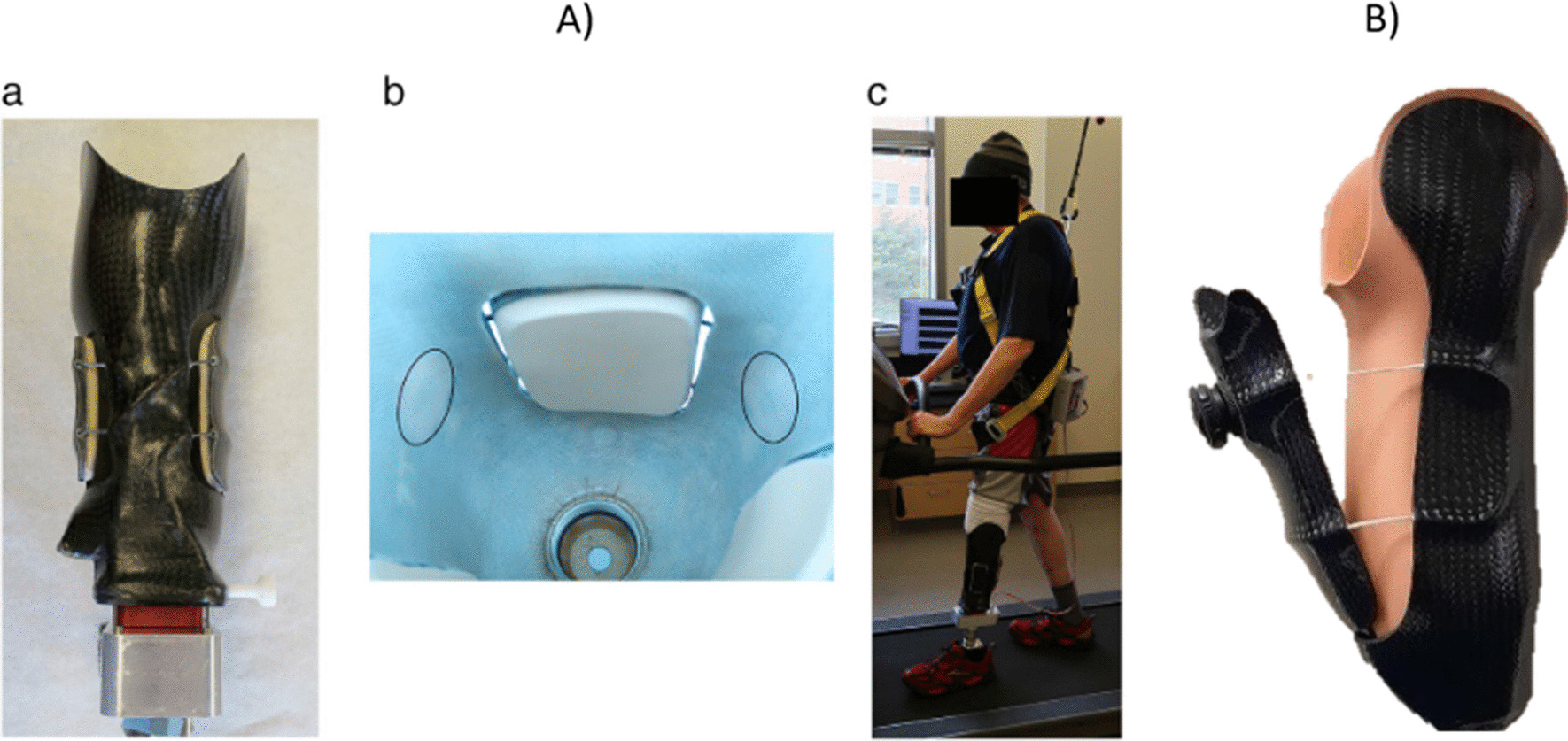
Examples of socket designs incorporating the moveable panel principle of adjustability. **A** A floating panel design by McLean et al. [35]. **B** A hinged panel design

Movable panel sockets are mostly rigid in form with certain sections able to move relative to the rigid section. There are two subcategories—floating (Fig. 4a) and hinged panels (Fig. 4b). Floating panel designs are usually classified as having a rigid surface form due to their material composition, with multiple DOF of movement due to their ability to accommodate small translations or rotations. Rigid single DOF panels, which can be floating or hinged, are only free to either translate along or rotate about one axis. How the panel is attached to the rigid section of the socket can determine which category it falls under and influence what form the panel surface may have. The surfaces of the moveable panels are built up with thicker panels enabling a greater reduction in socket volume (Fig. 4a). The material used is usually a firm foam to generate compression of the residuum, however if a softer material was used then the surface form could be classified as conformable.

In moveable panel designs, where the adjustment is local rather than across the whole socket, adjustment locations were generally justified based on load tolerant regions of the residuum or specific prosthetist advice. Lower limb sockets typically included either a single large posterior adjustable section, or three smaller adjustable sections (para-tibial and popliteal regions/ anteromedial, anterolateral, and posterior midline). For upper limb sockets, adjustable sections were placed at optimal myoelectric control locations. When a design has multiple panels they can either be adjusted by a number of actuator mechanisms, i.e. independently of one another [21, 32–34], or by a single actuation mechanism [33, 35–37]. Using a single actuator mechanism reduces the number of components required and the complexity of socket manufacture, however this creates an underactuated system where the same repeated input could create different internal socket geometries.

From the 14 studies of moveable panel sockets, the median number of participants was 12, with one article using a single participant. In addition to these studies, three articles detailed no participant involvement. In literature, the rationale behind moveable panels changes depending on the anatomical location of the socket. Ten studies concentrated on residual limb fluid volume retention and recovery (all in transtibial populations), with only three concentrating on volume fluctuation accommodation (transtibial, transradial and transfemoral) or electrode contact (trans-humeral).

##### Circumferential adjustment

**Fig. 5:**
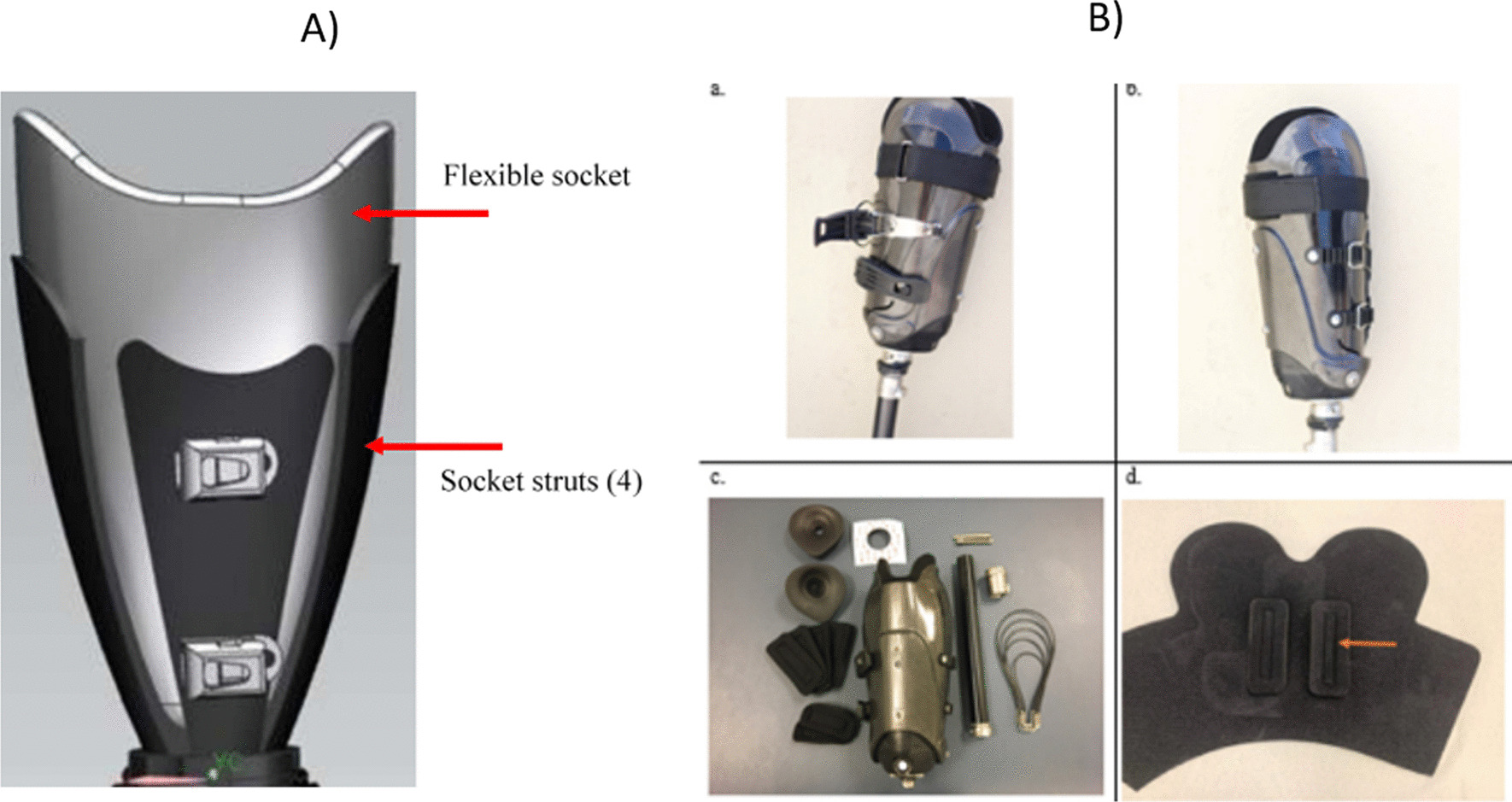
Examples of socket designs incorporating the circumferential adjustment principles of adjustability. **A** A strut design by Terrazas Quezada [46]. **B** An Overlap design by iFIT Prosthetics LLC [44], images from Kenia et al. [41]

The circumferential adjustment designs are characterised as either ‘gap/overlap’ or ‘struts’, with both being adjusted by straps or wires which travel circumferentially around the socket wall. Gap-based designs [38–40] and overlap designs [41–45] work by having an area of the socket, which when adjusted allows the circumference to change (Fig. 5b). The control mechanism for adjusting the socket is often placed over this area, making its location determined by user access.

Strut-based designs [46–50] consist of multiple longitudinal struts, often four, where each strut can flex/move independently (Fig. 5a). Some designs have the struts located against the liner to act like a conventional socket wall, whereas others allow for a gap between the struts and the residual limb, with contact only at the proximal end of the socket where the adjustment straps are located. Two socket designs [46, 50] can change the diameter of the socket at the distal end specifically by changing the radial distance between the longitudinal struts at their connection to a distal end plate, as well as providing more general circumferential adjustments throughout the socket’s length. For both designs, the distal end plate radius is changed manually, with the socket doffed, by the prosthetist.

Of the 18 articles covering circumferential socket designs, 13 involved participants, with a median number of six. Seven of the studies were conducted on transfemoral socket designs with the rest being a range of other anatomical locations. Accommodating volume fluctuations and the associated comfort of achieving this were the motivations behind most of the studies. Nearly 80% of the studies focusing on industry socket designs were clinical studies of circumferential socket designs.

##### Variable length

**Fig. 6:**
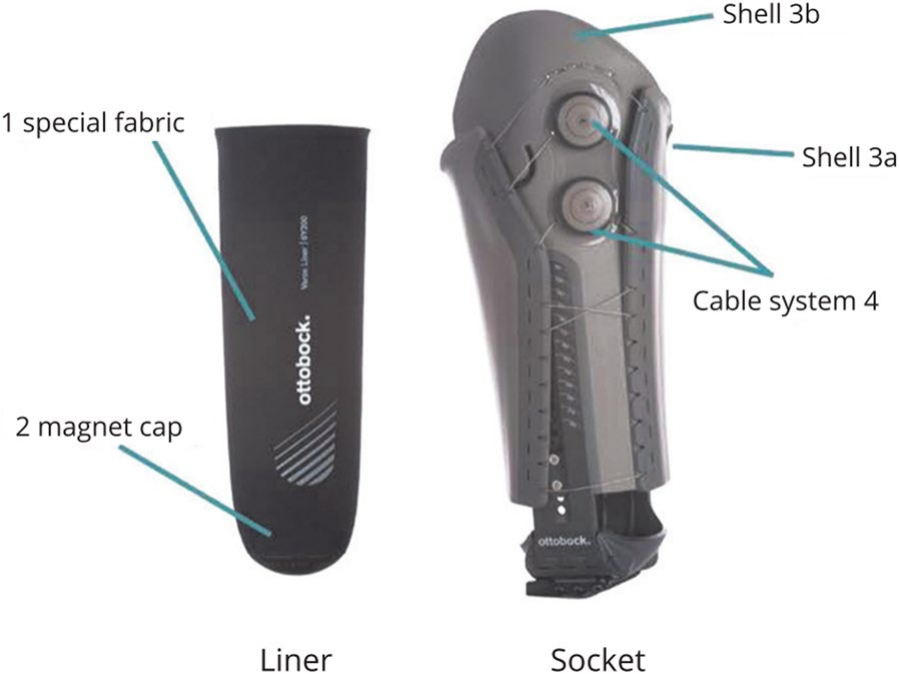
Example of a prosthetic socket including the Variable Length principle of adjustability. This socket is made by Ottobock [45], image from Nia et al. [42]

Socket length adjustability was only identified alongside circumferential adjustments. The variable length design by Hallworth et al. [51] is fully modular and the actuation mechanism for adjusting the socket volume can move longitudinally along the socket wall. As well as changing the socket length, this gives additional freedom in dictating where actuation and maximum compression may occur. The Connect TF [52], which is only clinician adjustable, uses the overlap technique to provide both its circumferential and length adjustments meaning the socket still encapsulates the residuum whereas the Varos socket [45] (Fig. 6) appears to just move the distal end connection away from the bulk of the socket. Finally, the LIM Innovations Infinite Socket TF [50] has an adjustable ischial seat height, varying part of the devices length, which is adjustable by prosthetists.

#### Surface form

Considering the designs according to the surface which is moved into contact with the residuum when the socket is adjusted, these can be characterised as conformable, rigid with multiple DOF, or rigid with a single DOF. Both literature and industry have shown very similar distributions by surface form (Table 2). However, use of inflatable bladders to deliver a conformable surface form was much more commonly seen in academic papers (89%) than in industry (17%). In industry, the additional conformable surface form designs were upper-limb circumferential adjustment sockets, consisting of fabric- and/or strap-based designs. However in literature only one upper-limb conformable surface design, categorised as a moveable floating panel design [53], and one lower-limb design, which is circumferential adjustment [54], were found. Only one design was found in literature which used circumferential adjustment specifically for upper limb and this had a rigid surface form with a single DOF of adjustability [51]. All the rigid multi-DOF surface forms fall within the moveable panels principle of adjustability across both literature and industry.

### Design variations

One research device by Ogawa et al. [55] used a magnetorheological fluid, which displays increased stiffness when exposed to a magnetic field, enabling the socket to have variable stiffness characteristics. This system resembles a device with fluid filled inflatable bladders, but when the magnetic field is applied the socket has the same characteristics as a rigid socket. Similar to this, the design discussed by Ibrahimi, Gruppioni and Menciassi [56] consists of an inflatable bladder built into the socket wall between two layers of jamming chambers. The jamming chambers are soft when at atmospheric pressure and become stiffened when the air pressure inside is reduced by vacuum. By pressurizing the different layers in order, the local stiffness characteristics of the socket wall can be changed allowing the socket to reduce in internal volume. Once reduced in size, the innermost jamming chamber is vacuumed to enable the socket to increase in stiffness and retain its new shape. Only one research device [57] combined moveable panels and circumferential adjustment into one concept. In this design the moveable panels are automatically adjusted using C-shaped shape-memory-alloy to provide a constant force by the panel, with the circumferential adjustments available to adjust the socket to suit the residual limb shape and size.

The two most recent patents are for socket designs that are a mesh of small, repeated components with specified tension between them, which claim to allow the socket to deform around the residual limb as it changes shape [58, 59]. This approach differs from the gap designs discussed above as there is a gap between each individual component rather than just at one location, but these could still be classified under circumferential adjustment.

### Actuation mechanisms

**Fig. 7:**
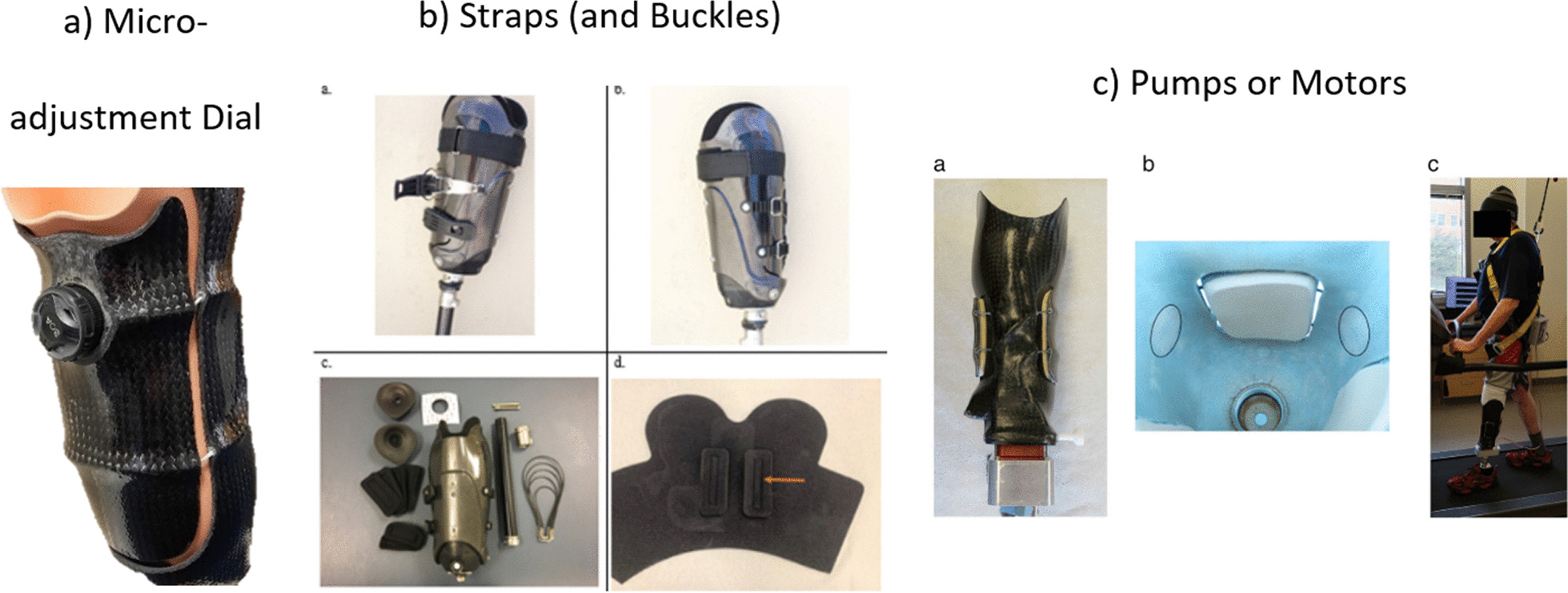
Examples of control mechanisms, **a** micro-adjustment dial, **b** Straps and Buckle [41], **c** motor [35]

We identified three main actuation mechanisms used across literature and industry: micro-adjustment dials, straps, and pumps or motors (Fig. 7). Some designs, including syringes used to increase/decrease fluid bladder volumes, and external motor or pump systems to control socket volume, were not practical outside of a laboratory environment. External control hardware in research-based devices is often bulky, potentially compromising cosmesis and device robustness. For this reason, any method of actuation deemed to be impractical to be used in everyday life was removed from this section of the review.

#### Micro-adjustment dials

Micro-adjustment dials are used to control cables which pass through the socket and adjustable regions. The most common design is the BOA dial [60] although other devices were also identified. These systems provide near continuous adjustment due to small increments of cable shortening when the dial is turned. All the commercially available designs which use cable driven control are manually adjusted by the user [25, 45, 52, 61].

#### Straps

Straps are generally positioned laterally around the external circumference of the socket and adjusted manually by the user. Some strap designs, such as those which utilise Velcro, provide continuous levels of adjustment whereas other systems such as leather straps or buckles [44] provide discrete levels of adjustment. The continuous systems, by nature, provide infinitely more adjustment levels over discrete systems, however discrete systems provide greater repeatability in setting adjustment levels. In lower limb research, Velcro straps appear to have been replaced by buckles to accommodate greater loads due to weightbearing, whereas industrial upper-limb sockets still use Velcro straps.

#### Pumps or motors

Pumps are used to control bladder-based systems and can provide a continuous range of adjustment by adding or removing air or liquid to one or more bladders. Some pumps also utilise control systems which are set to discrete levels predefined by a prosthetist based on specific socket pressures or limits, or even utilise automated control to maintain a predefined internal pressure level. Motors work similarly and can be used to pull panels towards the limb [35, 36, 62]. In these designs, the motor simply replaces the micro-adjustment dial and can be controlled remotely by either the user or researcher. Alternatively, with the inclusion of sensors to monitor the limb to panel distance, the motor can be controlled automatically to maintain socket fit throughout limb volume fluctuations [37].

### Cosmetics

From all the literature results only three articles [38, 39, 63] mentioned covering the adjustable socket with a cosmesis. All three studies involved participants wearing their adjustable socket outdoors which could explain their inclusion of a cosmesis, and interestingly these were also three of the earliest of the identified studies. With adjustable sockets available in industry, a similar distribution can be seen with only two companies [20, 64] mentioning the option of covers, both offering a choice of colours and designs.

### Other results

A range of methodologies were utilised to test the sockets and their principle of adjustability. When trialling lower limb devices, typical assessments such as the two-minute walk test and cycles of sitting and standing were used. These were predominantly carried out in controlled environments, such as a gait lab, and the studies typically examined the influence of the fit of the socket on the residual limb fluid volume. The assessment of upper-limb sockets focused more often on range of motion, mechanical performance and the ability of the socket to achieve good contact between an EMG (electromyography) sensor and the residuum (in designs intended for EMG controlled prostheses).

Only 17.1% of research designs focused on upper limb prosthetics, compared to 37.5% of commercial devices. None of the identified literature investigated both upper and lower limb adjustable sockets in the same study. Only one company [65] offered design concepts available for both the upper and lower limb, however, 47% of companies designed adjustable sockets for multiple lower limb amputation levels (e.g., transfemoral and transtibial sockets). In lower limb adjustable sockets, transtibial design are more prevalent than transfemoral in literature, 48.6% and 25.7% respectively, whereas the split is more even in industry, 43.8% and 50%.

*.*

**Fig. 8:**
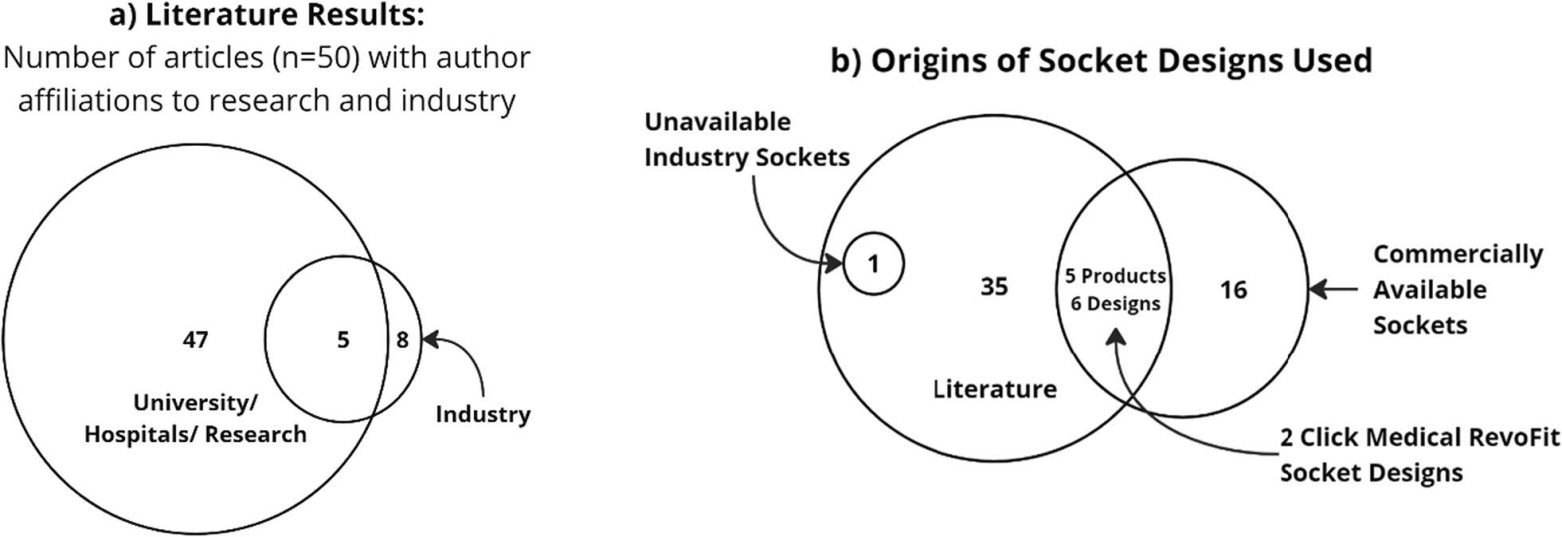
Plots indicating a lack of collaboration between research institutions and industry

Most prevalent in literature (77% of designs) were custom sockets, which are made to match the patient’s residuum shape without premanufactured component restrictions, likely due to the lack of industry partnerships present. In contrast, only 25% of industry designs are categorised as custom, however it must be noted that this is representative of the number of designs available and potentially not an indication on the quantity of sales or patients who use the devices. Only nine research articles cover the five different commercially available sockets used (Fig. 8). Two papers used the Click Medical RevoFit system [25], which is built into a custom socket, to build different designs of adjustable socket, with others appearing to use similar systems. This leaves 68.75% of identified commercial designs not associated to any identified research. One article by Greenwald, Dean and Board [66] discussed an inflatable bladder socket design with links to an industry company. The same socket design and associated industry connection was also made within the patent search, however no active product could be found.

The World Health Organisation (WHO) provide guidance on how research evidence should be interpreted to inform clinical decisions [67], referring to the hierarchy of internal study validity proposed by Murad et al. [68]. In prosthetic research, higher levels of evidence such as randomised control trials, and blinded studies, are known to be scarce [69]. The WHO however does recognise the need for lower-level studies to answer certain research questions. Of the 34 studies identified in this review, 18 arewere case studies, seven repeated measure studies, eight are cohort studies, and one did not disclose enough information to be categorised (Additional file 1: Appendix Table S3). Repeated measure studies are used over case control studies as they reduce the number of participants required, which is a known difficulty which limits prosthetic research.

## Discussion

This discussion addresses the design characteristics of the sockets, justification for the adjustment, and the limitations and safety of each design. The clinical studies were analysed to assess their scope and outcomes, both as an overarching assessment of adjustable sockets and in each principle of adjustability group. The control methods used are discussed, as well as the importance of cosmesis in design.

### Design justification

With patents and commercially available products, it is difficult to establish any justification behind design decisions as the content presented is either focussed on covering as broad a set of possible embodiments as possible (patents) or advertising the features and associated benefits of a product. In both cases, we found very few associated research publications. Interestingly, the information available through the Click Academy [40] describes how various design approaches may present different benefits to the user, however the research behind these claims is not presented. Instead, clinicians can share their custom designs for individual case studies, which provides some clinical evidence, but from small sample populations. Some companies [44, 64, 70] present research on their websites, some of which appeared in our literature results [41, 43, 49, 71]. Ottobock also have a clinical research area to their website [70], but the paper describing a clinical study of their socket [42] could not be found through it.

It was common across all adjustable socket designs for research studies to include clinical expertise to judge whether the initial socket shape had too tight or loose a fit, or to alternatively begin by duplicating the shape of the participant’s current clinically manufactured socket. These two options are likely favoured as there is limited research to guide decisions around socket fit. Several papers discussed creating a metric or pressure measurement when the socket had a good fit, often based on a prosthetist’s assessment, and then attempting to maintain this through adjustment [28, 66, 72]. An alternative method involved obtaining an approximation for the pressure limit of the residuum tissue, through indention testing [73] or numerical modelling [74], then restricting adjustments to that limit. It is also worth mentioning that using interface pressure alone has been shown to be a poor predictor of tissue damage, as similar interface pressure magnitudes often translate to considerably different internal tissue loads. This depends on factors such as the presence of bony prominences, and soft tissue characteristics [75], which can vary in amputee populations where comorbidities such as diabetic muscle infraction and neuropathy are more prevalent. Despite this, articles relating to inflatable bladders often justified their design through their ability to maintain a constant internal socket pressure [28, 66] or developed an algorithm which adjusted the bladders automatically in specific regions of the residual limb, depending on the pose of the arm (detected by an accelerometer [72]). Articles involving moveable panels rarely justified their design approach. In our opinion, moveable panels are the easiest principle of adjustability to standardise in manufacture and can be quantifiably evaluated, which could explain the popularity of their use within research and clinical practice.

### Socket adjustment

The range of adjustment made was most commonly reported as the percentage change in socket volume. Some articles referenced the estimated limits of a “good” and “acceptable” fitting socket, which are 5% and 10% change in socket volume respectively [76]. However, we did not find any research which compared the effects of varying the volume locally, as seen with moveable panels, or varying the volume generally, on the quality of fit, socket performance, as well as comfort and tissue health. This may well be dependent upon shape and tissue composition of each individual’s residual limb. Other studies had the range of adjustment determined by the participant themselves.

Small user adjustments are designed to accommodate low-level residual limb volume fluctuations or to increase the ease of a task, such as donning the socket [26, 44, 45, 61, 77]. Clinician adjustments are usually designed to alter the socket geometry to accommodate larger physiological changes without needing to redesign the socket or replace major components [46, 47, 51]. Together, these enable off-the-shelf and modular prosthetic sockets to be feasible, with one device potentially being suitable for a range of patients with varying residual limb sizes and shapes [15].

### Principles of adjustability

One limitation is that no sockets were purchased for physical inspection by the authors for the purpose of this review, therefore some assumptions on the details of how socket adjustments are achieved have been made, due to the limited detail provided within the literature and patents.

The proposed benefit of using inflatable bladders over alternatives is that they can control interface pressures, rather than volume, and can focus on local areas or the whole residuum. In order to achieve a consistent internal socket pressure, some designs consisted of multiple connected bladders covering the majority of the socket’s internal surfaces [28, 73, 78, 79], whereas others placed the bladders over specific load tolerant areas [66]. The evolution of the Epoch Medical design shows the relative difficulty in incorporating the required control system to automatically adjust an inflatable bladder into a conventional socket without adding weight. Such designs are also relatively complex, and hence introduce an additional risk of failure.

Moveable panels are cut to size, often from a fixed geometry socket, which ensures that the rest of the socket achieves a good fit, and only the load tolerant regions chosen are adjusted to accommodate limb volume fluctuation. The potential influence of panel size and locations on comfort and the mechanical coupling performance of the socket is unknown, with none of the studies investigating or discussing the relationship between coupling, comfort, and functionality. Adjustable sockets are well suited for investigating this due to the ease and relative repeatability with which the panel characteristics can be varied and adjusted to change the fit of the socket.

When considering underactuated moveable panel systems, it is unknown whether the panel with the least resistance (i.e., lowest interface pressure) would move when the socket is adjusted or whether the internal friction of the system from the wires would make panels closest to the dial actuate first. The actuator to panel ratio and its influence on adjustments is an area requiring future research, as an underactuated system could provide the freedom for the socket to adjust where needed.

Some designs presented through the Click Medical Academy [40] show how moveable panels can be used to aid the donning process and socket suspension, particularly with sockets intended for joint disarticulations where the distal aspect of residual limbs can be more bulbus. This demonstrates the potential versatility of this design compared to others.

In circumferential adjustment gap/overlap designs, the flex in semi-rigid socket material allows the socket to change shape. As a result, the control mechanism on the socket can only provide a force to reduce the socket volume from its manufactured shape, any flex in the other direction would be driven by an internal socket force to increase the effective volume. In strut designs, due to the ability of each strut to move independently of one another, the change in socket volume might occur where the residual limb has changed shape or where the residuum can accept additional compression. However, these struts are quite stiff, limiting the amount they can conform to the shape of the limb. Strut designs, where there is space between the struts (Fig. 5a), allow for small volume fluctuations of the limb to occur without needing to adjust the socket and maintain a consistent socket fit, meaning the socket can cope with a larger range of volume changes than other designs. This makes them ideal for residual limbs which see large daily volume fluctuations. However, this increases the potential for the socket to have areas which are overtightened, which could lead to discomfort or tissue damage, or over-loosened, which could adversely affect the mechanical coupling and functionality. One study reported a small drop in comfort after completing a mobility test, when compared to their current socket, as the single participant could feel the struts flexing whilst ambulating [46].

As discussed, variable length was only available alongside other principles of adjustability. This is particularly useful when using adjustability to make off-the-shelf sockets viable for low resource settings. The clinician can set the length and circumference of the socket to specific dimensions based on the geometry of the patient’s residual limb, with the user being able to only make subtle adjustments away from this setting, reducing the need for custom socket design and manufacture. This is particularly effective with upper-limb socket design, due to the relatively smaller residual limb volume fluctuation, with examples of this seen in commercially available devices from Martin Bionics (Socketless Socket) and Toughware (ITAL) [23, 65]. Despite these designs existing only the Toughware mentions low resource settings, likely due to the majority of funding in prosthetics coming from high resource countries.

### Safety and limits

Due to the low number of inflatable bladders designs, the safety of these devices is difficult to evaluate. However, this principle of adjustability could be considered safer than other devices with respect to tissue loads seen, due to the constant pressure nature of liquid- or air-filled bladders. On the other hand, the risks associated with the bladders either leaking, bursting, or delaminating from the socket, are unique to this type of device.

Although in research studies of liquid-filled inflatable bladders, liquid can be added from an independent reservoir, practical designs are limited by the amount of liquid that can be stored on the prosthesis. Air bladders show greater potential as they can operate without the need for a reservoir. In both cases, the socket shape can only be reduced from an initial geometry, meaning the sockets are cast and manufactured at maximum residuum volume for the intended user, or the socket design would be restricted to accommodating limb volume reduction only. Further to this, whilst it is feasible to change the volume of the bladders occasionally throughout the day, the designs which require the bladder volume to adjust during the gait cycle are limited by the combination of the fluid’s viscosity and the pump flow rate.

Moveable panels and circumferential adjustment gap designed sockets have a visually obvious and defined minimum volume for the socket. In moveable panel designs, the panel thickness can be customised to control the limit on socket volume reduction, which can be seen by the user as the panel becomes flush with the rigid socket. Likewise in custom circumferential adjustment gap designs, the gap shape and size can be personalised to suit the individual’s range of residuum volume fluctuations. As this gap closes, the material stress is theoretically dispersed throughout the socket wall, however with the nonuniform shape of a socket wall and uneven internal pressures provided by a residuum, this is unlikely the case and makes it unclear whether adjustment of the socket affects local or whole socket geometry. This suggests that these sockets have less control over the specific location that socket volume change occurs when compared to other principles of adjustability, potentially causing localised discomfort and tissue damage. However, this could design some freedom into the socket to function as an underactuated system, adjusting the socket shape where required. Further research is required into circumferential sockets to understand how the socket shape and location of the gap/overlap influence where the socket volume is adjusted. Designs which offer localised adjustment rather than whole volume, such as moveable panels, could be safer than circumferential adjustment devices due to their ability to focus load on load tolerant regions and avoid areas which are sensitive to loading and at risk of tissue damage.

### Clinical studies

The low participant numbers perhaps reflect the relatively early stage of this field’s development and may also reflect the relatively small population of eligible research participants, particularly those with upper limb absence.

The upper-limb study tests were exclusively carried out in controlled environments (such as a laboratory), and rarely examined the impact of adjustment once the socket was fitted, probably due to the upper limb experiencing much less volume fluctuation when compared to lower limbs [80]. Importantly, studies were often short-term in duration regardless of whether they focussed on upper or lower limb adjustable sockets, greatly limiting the extent to which the safety and durability of these devices could be tested.

In the four studies of inflatable bladder devices, which included multiple participants, each had different aims and outcome measures, reducing the ability to extrapolate the findings. Some concentrated on residual limb fluid volume and recovery and others focussed on residuum-socket interface pressure. One study [79] found that increasing bladder volume and therefore interface pressure reduced residual limb volume in a high proportion of participants. Interestingly, optimal self-reported comfort across studies was associated with different bladder pressures between participants, highlighting the subjectivity involved with obtaining comfort data.

Higher participant numbers in moveable panel studies could be related to the ease with which moveable panels can be incorporated into a prosthetic socket. Some studies showed that moveable panels can effectively aid in the retention of residual limb fluid during exercise and rest [32, 33], however these studies used motors to positively detract the panels to facilitate this recovery. Whilst effective in illustrating that the resulting vacuum is beneficial to the user’s tissue health, conventional actuation mechanisms do not offer this function. This is therefore an example of adjustable sockets being designed to test a research question rather than as a commercially viable design. Very few moveable panel studies included an outcome measure of socket comfort, despite this being the major outcome measure used in UK NHS clinics. Those few papers that did report comfort were inconsistent in their methods, with some comparing between adjustable settings and others comparing to previous socket designs. One study [34] included a subjective measure allowing participants to comment on why the comfort changed between adjustment settings. The range of comments shows that there is a need for a more in-depth socket comfort outcome measure to be established to allow consistent reporting on how different adjustable socket designs influence participant comfort. In contrast to the studies on inflatable bladders and moveable panels, over half of the studies on circumferential adjustment sockets included a verified and established method for assessing comfort. The majority of these reported improvements in comfort when using the adjustable socket over the participants’ original/current sockets, however no reasons are documented as to why this occurs. Future studies should assess how comfort changes between, and whilst set at, different levels of adjustment, as well as the effect of the location of adjustment on comfort.

Further outcome measures used across the clinical studies included bioimpedance measures of residuum fluid volume, internal socket pressure measurements, and subjective comfort and pain scores, but the use of these varied greatly from study to study making it difficult to compare the designs used in different studies, particularly as no clinical study compared different designs of adjustable socket. Many of the studies also used the internal socket pressure to inform how tight the socket was fitted, which may also have been a factor in improving the socket comfort, as it prevented the socket from being overtightened in order to achieve a better mechanical coupling between the residual limb and socket. The literature was less clear on whether an adjustable socket improved gait. If adjustments were controlled to ensure comfort, with pressure kept to a reasonable level, it is possible that observed changes to gait could have been driven by improved comfort. The relationship between changes to coupling and gait are difficult to study without careful study design and hence there is a need for further research investigating how an adjustable socket affects both comfort and coupling, and the challenges in achieving a compromise between them.

The Medicines and Healthcare products Regulatory Agency (MHRA) recognise prostheses are made up of two separate parts: the prosthetic socket, and the hardware (everything else). They stipulate that prosthetic sockets can be either a Class 1 medical device in their own right, or custom made by a clinician to manufacturer guidelines [81]. Despite some adjustable prosthetic sockets being Class 1 medical devices, and many likely having the potential to cause harm if improperly adjusted, of the 34 research studies involving participants only five reported on adverse events. The short-term nature of studies may also be contributing to a potential under-reporting of risks.

### Actuation mechanisms

#### Micro-adjustment dials

The maximum tension within the cable, and therefore pressure applied by adjustable sections, is limited by the strength of the cable and dial used, which are not visible to the user. There is therefore the potential for the user to continue tightening until the socket reaches its minimum designed volume. Another disadvantage of these dials is that when mounted to the external surface of the socket, they stand proud compared to the rest of the socket, compromising cosmesis (Figs. 4b and 7a). The dials could also catch on clothing or other objects, causing damage and affecting function. Often designed to work with laminated sockets to protect the cables, micro-adjustment dials may only be viable in clinics where lamination capabilities are available.

#### Straps

Both continuous and discrete strap mechanism can be easily designed to include restrictions to prevent the user from over or under tightening their socket, as can be seen on the system used by iFit Prosthetics [44] which provides discrete levels of adjustment. The disadvantage of Velcro is that any adjustments require the Velcro to be completely undone before being reapplied, making small incremental changes more difficult.

Out of all the control mechanisms discussed, straps are the easiest to implement as they can be manufactured from materials already used in prosthetics and orthotics clinics and are cheap to purchase. This factor is particularly important for clinicians working in low resource settings. Furthermore, straps have a relatively low profile relative to the socket compared to the other two control mechanisms (Fig. 7). Straps raise few safety concerns as a control mechanism. However, continuous strap systems have the potential to slip when under higher forces and straps made from leather or polymers/plastics may stretch and break over time.

#### Pumps or motors

Pumps and motors enable the clinician to provide specific limits to adjustments, preventing the user from overtightening or loosening their adjustable socket. This is a key safety feature when utilising this actuation mechanism as the potential range of adjustment without any form of restrictions could be quite large.

The disadvantage of these actuation mechanisms is that the additional components need to be positioned somewhere on the prosthesis. Epoch Medical [30]; Greenwald, Dean and Board [66]; and Washington University [35–37, 62], all position these at the distal end of the socket along with the reservoir of additional fluid or cable (Fig. 7c) adding bulk and weight to the prosthesis. Its distal positioning increases the inertia of the limb, potentially affecting gait and the ease with which the limb is controlled. Due to the size of these components, these designs may not be feasible on trans-tibial or trans-radial prostheses for individuals with longer residual limbs where space is limited. As the control mechanism is mounted away from the adjustable locations, the fluid or cable routings also need to be included into the prosthetic socket, further complicating the design and manufacturing process.

### Cosmetics

A cosmetically pleasing or inconspicuous prosthesis can be a key factor in increasing prosthesis satisfaction and embodiment [82].The majority of more recent clinical studies were lab based and it appears the issue of cosmesis was of less importance when compared to the need to observe and interact with the socket and its adjustable components. The low number of companies offering covers is surprising considering that these products are intended for everyday use. This lack of covers, or cosmeses, is likely down to the need to access the control mechanisms which are positioned on the external socket wall, along with the cover needing to adapt to the changing socket shape. This is avoided on the LIM Innovations TT-S [61] socket as the cover is loosely fitting and the adjustment dial has been lowered away from the socket wall, so that it is outside of the cover. The lack of covers could also be due to the increased number of aftermarket covers, or wrap-arounds, becoming available, providing a bespoke appearance.

### Patent search methodology

Although 73% of the companies were discovered through the patent search, over 60% of the patent results could not be linked to either companies with active products, or to research institutions, indicating that the patent search criteria could be further refined in the future to identify relevant and appropriate patents more accurately within this field. Using the same search criteria for both the literature and patent searches would have reduced its effectiveness further as patents are often given more generic names than research articles; an example of this is the increased use of the word ‘system’ in patents over literature.

## Conclusions

The adjustable socket, whilst not a new concept, is gaining popularity, as shown in the recent increase in the number of publications, patents, and commercially available designs. This review identifies four principles of adjustability (inflatable bladders, moveable panels, circumferential adjustment, and variable length) and three surface forms (conformable, rigid with single DOF, and rigid with multi-DOF) which define a new method of classification which better informs how the socket volume is adjusted. This classification can be combined with the manufacturing-focussed classification used previously (off-the-shelf, modular, and custom) to further describe socket characteristics. There is a clear difference in design approaches used commercially compared to those being investigated in research, with commercial designs often using one or more panels or circumferential adjustment to provide adjustment, whilst research often focusses on bladder-based concepts. Reporting of justification for the location(s) and range of adjustment provided has been found to be lacking across both commercial and research sockets. Some principles of adjustability limit the minimum volume a socket can reach; however, straps and pumps are the only actuation mechanisms that can contain safety features which limit the range of adjustment, preventing over, or under-tightening of the socket and reducing the potential for either tissue damage or, in extreme cases, preventing the socket from falling off. Safety is often neglected from articles describing adjustable socket designs.

Adjustable sockets, especially off-the-shelf devices, also have potential when considering low resource settings. Supplying users with sockets which only require minor modifications from the clinician to achieve a good fit, with smaller adjustments available to the user, is an effective method of reducing initial fitting time and frequency of visits for the patient. Despite this few designs explicitly mentioned low resource settings and only one study involving adjustable sockets had been carried out in a low resource context [41]. There is a need to prioritise research into the suitability of adjustable sockets globally, particularly in low resource settings, as these could effectively reduce the cost, resources, and time currently used to fit and maintain prostheses.

This review is subject to several limitations, firstly the review only focussed on papers which were available in English or French. Secondly, the majority of clinical studies were restricted to small case or cohort studies, likely due to the difficulty in accessing participants with limb difference. Hence, any real-world testing of the adjustable sockets was limited in its extent. The literature found also didn’t focus on the negative trade-offs that users may experience through using adjustable sockets, such as the relative cost of such devices and the increased complexity (and therefore increased risk of failure or malfunction).

Future work should also focus on improving the exchange of knowledge between researchers and industry, with it being noted that eleven of sixteen commercial designs identified (68.75%) were not supported by published clinical studies. Using scientific methods to analyse these designs would be beneficial to understanding the effectiveness of each design group, and what patient sub-groups could benefit most from each design, as well as potentially leading to improved commercial designs and more relevant clinical studies. Furthermore, across all studies there was little to no investigation or discussion regarding what quantifies a well-fitting prosthetic socket, and this is an area of research which is currently lacking and needs to be understood to help inform future socket design. To achieve this, it is suggested that future work could investigate the relationship between socket design, mechanical coupling, comfort, and residual limb tissue health. Finally, adjustable sockets could be utilised effectively to analyse this due to their ability to alter their coupling with the residual limb with relative ease and repeatability.

### Supplementary Information

**Additional file 1.** File details patent, industry, and literature results identified with socket, and study, characteristics listed.

## Data Availability

No new data were generated or analysed during this study.

## Abbreviations

DOF: Degrees of freedom
NOFT: Everywhere except the full text
PRISMA: Preferred Reporting Items for Systematic Reviews and Meta-Analyses
EMG: Electromyography
MHRA: Medicines and Healthcare products Regulatory Agency
WHO: World Health Organisation
