# Supplementary material for: Adjustable prosthetic sockets: a systematic review of industrial and research design characteristics and their justifications
Source: J Neuroeng Rehabil. 2023 Nov 6;20:147. doi: 10.1186/s12984-023-01270-0 (PMC10626671; doi:10.1186/s12984-023-01270-0)
Supplement: Supplementary file 1 — Additional file 1. File details patent, industry, and literature results identified with socket, and study, characteristics listed. [file 12984_2023_1270_MOESM1_ESM.docx]

# Appendix – Search Results Tables

## Patent Results

*Table S1: Patents linked to companies or university institutions.*

|  | Company | Year Published | Patent Number & Reference | Patent Name |
| --- | --- | --- | --- | --- |
| Current Products Available | CJ Socket Technologies | 2012 | US2012041567A1  ([1](#_ENREF_1)) | Adjustable prosthetic limb socket |
|  | Click Medical | 2011 | US2011071647A1  ([2](#_ENREF_2)) | Adjustable prosthetic interfaces and related systems and methods |
|  | Epoch Medical | 2022 | US2022110768A1  ([3](#_ENREF_3)) | Modular adjustable prosthetic volume compensation system |
|  |  | 2019 | US10357382B2  ([4](#_ENREF_4)) | Adaptive compression prosthetic socket system and method |
|  | Flo-Tech | 1998 | US5728165A  ([5](#_ENREF_5)) | Adjustable post-operative preparatory prosthetic system |
|  | IFIT Prosthetics LLC | 2012 | US2012259433A1  ([6](#_ENREF_6)) | Modular Prosthesis System |
|  |  | 2013 | WO2013056203A2  ([7](#_ENREF_7)) | Above-the-knee modular prosthesis system |
|  |  | 2022 | US11382775B2  ([8](#_ENREF_8)) | Modular prosthetic devices and prosthesis systems |
|  | LIM Innovations | 2016 | US2016058584A1  ([9](#_ENREF_9)) | Prosthetic socket with an adjustable height ischial seat |
|  | Martin Bionics | 2020 | US2020397602A1 ([10](#_ENREF_10)) | Air hammock for a prosthetic |
|  | Ossur | 2012 | US2012101597A1  ([11](#_ENREF_11)) | Adjustable socket system |
|  |  | 2020 | US10543112B2  ([12](#_ENREF_12)) | Adjustable prosthetic limb system |
|  | Ottobock | 2019 | US2019183663A1  ([13](#_ENREF_13)) | Prosthesis socket and method for controlling an adjustment of an inner circumference of a prosthesis socket |
|  | Stumpworx | 2021 | US2021298928A1  ([14](#_ENREF_14)) | Variable compliance prosthetic socket with breathable matrix |
| University Institutions | Washington University (US) | 2020 | US2020345520A1  ([15](#_ENREF_15)) | Motorized adjustable socket for amputee prosthesis users and methods for use thereof |
|  | Arizona State University (US) | 2021 | US11173057B2  ([16](#_ENREF_16)) | Volume adjustable transtibial socket |
|  | Clemson University (US) | 2015 | WO2015013560A1  ([17](#_ENREF_17)) | Prosthetic limb having an adjustable socket |
|  | Xi An Jiaotong University (CN) | 2018 | CN108451676A  ([18](#_ENREF_18)) | 3D printing prosthetic socket with adaptability |
| No Link to Current Product Found | Adjustable Artificial Limb Inc | 1917 | GB109085A  ([19](#_ENREF_19)) | Improvements in Artificial Limbs |
|  | BHN Incorporated | 1979 | US4161042A  ([20](#_ENREF_20)) | Adjustable Prosthetic Limb |
|  | Blatchford Products LTD | 2021  2022 | US2021113356A1  ([21](#_ENREF_21))  GB2606264A | Prosthetic Limb Socket |
|  |  | 1983 | GB2103490A  ([22](#_ENREF_22)) | Artificial Limb Socket |
|  | Easyfit Orthopaedics | 2015 | WO2015103506A1  ([23](#_ENREF_23)) | Dynamic Adjustable Socket for Prosthetics |
|  | Opengait | 2020 | US2020297514A1  ([24](#_ENREF_24)) | Modular, adjustable, above-knee prosthetic socket |
|  | OrthoBionics | 1989 | US4842608A  ([25](#_ENREF_25)) | Fluctuating volume adjustable preparatory below knee prosthetic socket |
|  | Ottobock Healthcare | 2014 | US2014243996A1  ([26](#_ENREF_26)) | Prosthesis Assembly |
|  | Scinetics | 2021 | US2021353441A1  ([27](#_ENREF_27)) | Modular Prosthesis System |
|  | Simbex | 2002 | US2002099450A1  ([28](#_ENREF_28)) | Dynamic variable geometry fitting system for use with a body appliance |
|  | VBionic | 2020 | WO2020078864A1  ([29](#_ENREF_29)) | An Adjustable Prosthetic Socket and Suspension System |
|  | Vivonics | 2016 | US9254200B2  ([30](#_ENREF_30)) | Active Prosthetic Socket |

*Where multiple patents were a part of the same patent family, the latest patent number only is listed and referenced.*

## Industry Results

*Table S2: Industry Products and Details.*

| Principle of Adjustability | | Company | Product Name | Location | Manufacture Classification | Adjustable Surface  Form | Method of Adjustability | Motivation | Design Characteristics |
| --- | --- | --- | --- | --- | --- | --- | --- | --- | --- |
| Inflatable Bladders | | **Epoch Medical**  ([31](#_ENREF_31)) | Unknown | TT | Custom | Conformable | Automatic | Accommodate volume change and manage limb fluid volume | Internal fluid bladder on posterior surface automatically controlled to retain socket pressure |
| Moveable Panels/ Floating Panels | | **Quorum Prosthetics**  ([32](#_ENREF_32)) | Quatro TM | TT, TF | Custom | Rigid – Multi-DOF | Manual | Daily volume fluctuation, Ease of don and doffing | 3D printed socket shape & panel locations, manual adjustment of 3 independent panels to adjust volume |
|  |  | **LIM Innovations**  ([33](#_ENREF_33)) | Infinite Socket | TT | Modular | Rigid – Multi-DOF | Manual | Accommodate daily volume fluctuations to improve comfort | Posterior proximal panel controlled by BOA dial |
|  |  | **Martin Bionics**  ([34](#_ENREF_34)) | Socket-less Socket | TT, TR, TH, | Modular | Rigid – Multi-DOF | Manual | Lightweight, dissipate heat, accommodate changing limb shape | Posterior strap that moves 2/3 posterior panels to adjust diameter |
| Circumferential Adjustment | **Struts** | **Martin Bionics**  ([34](#_ENREF_34)) | Socket-less Socket | TF, HD, SD | Modular | Conformable | Manual | Lightweight, dissipate heat, accommodate changing limb shape | Posterior strap that moves 2/3 posterior panels to adjust diameter |
|  |  | **Stumpworx**  ([35](#_ENREF_35)) | Stumpworx Arm | TR | Modular | Conformable | Manual | Full range of motion with comfortable, adaptable suspension | Collection of pads which interact with key bony landmarks, connected with an adjustable lacing which can be loosened and tightened as desired |
|  | **Gap/Overlap** | **CJ Socket Technologies**  ([36](#_ENREF_36)) | CJ Sail | TT, TF, TR, TH | Custom | Conformable | Manual | Limb volume change and comfort when sitting/bending, reduce prosthesis abandonment | Fabric section of the socket to allow for volume fluctuation |
|  |  | **FLO-Tech Systems**  ([37](#_ENREF_37)) | FLO-TECH TOR | TT, TF | Prefabricated | Rigid – Single DOF | Manual | Accommodate various limb geometry shapes and sizes and fluctuations | Proximal band that controls the overlapping of multiple segments of socket |
|  |  | **iFIT Prosthetics LLC**  ([38](#_ENREF_38)) | iFIT | TT, TF | Prefabricated | Rigid – Single DOF | Manual | Comfort, stability and durability throughout changes in limb volume | Customise liner to individual, 2/3 posterior straps to adjust socket |
|  |  | **KOALAA**  ([39](#_ENREF_39)) | ALX and PAWW | TR, WD, PH | Prefabricated | Conformable | Manual | Lightweight, comfortable, adjustable socket Children and primary limb loss | Plastic sleeve cut to length; Velcro/boa used to adjust circumferential compression/fit |
|  |  | **Ossur**  ([40](#_ENREF_40)) | Connect TF | TF | Prefabricated | Rigid – Single DOF | Manual | Ease of don and doffing. Clinician adjustable to accommodate limb size changes, remove refitting requirements | Posterior panels that tighten and loosen, don & doff handle for quick release |
| Circumferential Adjustment and Socket Length | **Struts** | **LIM Innovations**  ([41](#_ENREF_41)) | Infinite Socket | TF | Modular | Rigid – Single DOF | Manual | Accommodate daily volume fluctuations to improve comfort. Adjustable ischial seat for comfort. | TF additional prosthetist adjustment of socket length |
|  |  | **TU Delft**  ([42](#_ENREF_42)) | WILMER | TR | Modular | Rigid – Single DOF | Manual | Ease of don and doffing, fast and more adequate fitting, improved fixation, reduced perspiration | Upper arm brace can be moved proximally to create space for donning/doffing, upper arm brace can be locked to create a variety of socket sizes |
|  | **Gap/Overlap** | **Ottobock**  ([43](#_ENREF_43)) | Varos | TF | Prefabricated | Rigid – Single DOF | Manual | Quicker fitting, easy donning/doffing, adjustable volume | Dual dials to adjust lateral panel to adjust volume of socket Side rails to adjust length/position of socket relative to distal end |
| Circumferential Adjustment, Socket Length and Adjustable Elbow Cuff | **Gap/Overlap** | **Toughware**  ([44](#_ENREF_44)) | ITAL | TR | Prefabricated | Conformable | Manual | Air circulation, sweat drainage, fast fitting, lightweight, adjustable fit | Adjustable humeral suspension cuff and forearm shell which can be radially adjusted using straps |
| Panels and/or Circumferential Adjustment | | **Click Medical**  ([45](#_ENREF_45)) | RevoFit | N/A | Custom | N/A | Manual | Build in adjustability anywhere, Micro adjustments, reduce clinic visits, targeted compressions | Ratchet and wire-based system which can be applied in a variety of ways to adjust the geometry/volume of laminated sockets |

*(Locations: TT-Trans-tibial, TF-Trans-femoral, TR-Trans-radial, TH-Trans-humeral, HD-Hip Disarticulation, SD-Shoulder Disarticulation, WD-Wrist Disarticulation)*

## Literature Results

*Table S3: Literature Results and Details.*

| Principle of Adjustability | Reference | Manufacture Classification | Location | Adjustable Surface Form | Method of Adjustment | Motivation | Design Characteristics | Study Details | Participants | Study Outcomes | Study Type |
| --- | --- | --- | --- | --- | --- | --- | --- | --- | --- | --- | --- |
| Inflatable Bladders | [Candrea, Sharma (46)](#_ENREF_46) | Custom | n/a | Conformable | Automatic | n/a | Design: Concept only which consists of 4 circumferentially arranged air bladders  Control: Bladder volume can be altered using a pump.  Safety: Not Specified | n/a | - | n/a | n/a |
| Inflatable  Bladders | [Carrigan, Nothnagle (47)](#_ENREF_47) | Custom | TT | Conformable | Manual | Improve socket fit | Design: Small air-filled actuator inserts which can be positioned within any socket, between the socket and residual limb to vary the volume and shape of the socket.  Control: Actuator volume can be adjusted by adding/removing air via a pump and air pressure regulator.  Safety: Not Specified | Gait simulator used with a prosthetic socket monitored using pressure sensors and a 45.5kg weight. Gait was simulated over a 100 second time period. | - | The measured pressures were similar to those expected from the internal actuator pressures set. | n/a |
| Inflatable  Bladders | [Greenwald, Dean (48)](#_ENREF_48) | Custom | TF and TT | Conformable | Manual | Volume fluctuations | Design: Several bladders placed within the interior of the socket (number and position determined by prosthetist).  Control: Mechanical control circuit which pumps fluid in and out of the bladders from a reservoir.  Safety: Prosthetist sets maximum pressure, and the user can adjust within the set parameters. | n/a | - | n/a | n/a |
| Inflatable  Bladders | [Gu, Yang (49)](#_ENREF_49) | Custom | TH | Conformable | Automatic | Comfort & functionality | Design: 4 bladders arranged circumferentially around the interior of the socket.  Control: Each bladder is controlled independently, with air pumped in and out of them to adjust the socket. The level of inflation is determined using an algorithm which is based on the pose of arm (using an accelerometer) and readings from pressure sensors under each bladder.  Safety: Algorithm keeps the pressure at a reasonable level. | 1 healthy participant was involved in laboratory-based testing to provide a proof of concept. | 1 | Socket was comfortable and able to be worn without complaint for several hours. | Case Study |
| Inflatable  Bladders | [Ibrahimi, Gruppioni (50)](#_ENREF_50) | Custom | TF | Conformable | Unclear | Local shape adaption & pressure relief | Design: Socket contains actuators which are positioned on stressed anatomical areas. They sit between a rigid external socket and flexible internal layer. The actuators function using layer jamming chambers which combine vacuum or atmospheric pressure to create a material with variable stiffness that can conform to the residual limb.  Control: The arrangement and pressure of these chambers can be altered to modify the fit of the socket.  Safety: Not Specified | Workbench testing on artificial limbs. | - | Workbench testing showed proof of concept, with variable stiffness socket achieved. Future work will involve studies on participants with limb loss. | n/a |
| Inflatable Bladders | [Ma, Foca (51)](#_ENREF_51) | Custom | TT | Conformable | Manual | Volume fluctuation | Design: Socket contains a soft bladder which is filled with air.  Control: A valve at the distal end of the socket can be used to vary the volume of air within the bladder to vary the socket volume.  Safety: Not Specified | Been supplied to 15 elderly users who suffer from cardiovascular disease. | 15 | “Promising benefits” | Unknown |
| Inflatable  Bladders | [Mandić, Manev (52)](#_ENREF_52) | Custom | TT | Conformable | Manual | Volume fluctuation & shape changes | Design: Made of a flexible resin interior and a hard resin exterior, with the space between the two layers filled with liquid in the middle and air in the upper part. Control: A valve is used to add or remove liquid and air when needed. The use of incompressible liquid ensures that the pressure is evenly distributed across the residual limb.  Safety: Not Specified | n/a | - | Device does not surpass standard PTB prosthetic weight (AT TIME OF PAPER). Aesthetic appearance is satisfactory, and the socket is easy to operate. | n/a |
| Inflatable  Bladders | [Mbithi, Chipperfield (53)](#_ENREF_53), [Mbithi (54)](#_ENREF_54) | Theoretical | TT | Conformable | Automatic | Reduce soft injury risk | Design: Various designs of several adjustable actuators positioned at the patella tendon, medial and lateral tibial flares, posterior calf, mid-tibial crest and lateral fibula flare. These are either spring actuators or fluid filled bladders which provide either a rigid or soft interface with the residual limb.  Safety: Not Specified | n/a | - | n/a | n/a |
| Inflatable  Bladders | [Montgomery, Vaughan (55)](#_ENREF_55) | Theoretical | TT | Conformable | N/A | Volume fluctuation | Design: Concept design consisting of rigid outer shell with an inner membrane consisting of one large inflatable bladder.  Control: Not Specified  Safety: Not Specified | n/a | - | n/a | n/a |
| Inflatable  Bladders | [Razak, Osman (56)](#_ENREF_56) | Off the Shelf | TH | Conformable | Automatic | Improve comfort & reduce interface pressures | Design: Air splint (where the socket is solely the inflatable bladder).  Control: Force sensor at interface turns pump on until the interface pressure reaches desired target (around 10kPA).  Safety: | Participants with 12cm residuum and no requirement for assistive devices. Tekscan pressure sensor, test just involved setting up of prosthetic socket | 15 | Comfort increased regardless on scar tissue or bony prominences being present. | Case Study |
|  | [Abd Razak, Osman (57)](#_ENREF_57) |  |  |  |  |  |  | Interface pressures recorded in initial setup of Air Splint and polypropylene sockets | 1 | Air Splint reduced interface pressures compared to the polypropylene socket | Case Study |
| Inflatable  Bladders | [Sanders, Cagle (58)](#_ENREF_58) | Custom | TT | Conformable | Manual, laboratory only environments | Fluid retention | Design: Consists of 4 fluid filled bladders positioned within the socket at lateral tibial flare, medial tibial flare, posterior lateral, posterior medial positions.  Control: The volume of each bladder can be varied using a syringe.  Safety: Not Specified | The bladders were added to the interior of their own sockets. Participants would walk for 90s with the bladders full, then rest and walk for 90s with the bladders empty. For each cycle of the volume of each bladder was increased. | 19 | 15/19 of the participants experienced gradual residual limb fluid loss across the study. Reducing the socket volume may accentuate residual limb fluid loss but is not consistent. | Case Study |
| Inflatable  Bladders | [Sang, Li (59)](#_ENREF_59) & [Sang, Li (60)](#_ENREF_60) | Custom | TR | Conformable | Manual or Automatic | Comfort | Design: 4 circumferentially arranged inflatable chambers.  Control: Air can be pumped in and out of these chambers and discrete levels of adjustment can be set which can be switched between when appropriate. Safety: Indention testing of the residual limb tissue helped determine the maximum pressure value setting for the chambers. | Laboratory-based testing of the socket, switching between predefined states for the bladders. Healthy participants. | 6 | Improved comfort and function, combined with fewer issues regarding temperature and humidity. | Case Study |
| Inflatable Bladders | [Seo, Lee (61)](#_ENREF_61) | Custom | TT | Conformable | Automatic | Retain internal socket pressure | Design: Consists of 3 soft air bladders located on anterior and posterior tibia areas.  Control: Pneumatic valve and PID controller used to maintain constant internal socket pressure.  Safety: The pressure level is maintained to ensure excess pressure levels are avoided. | Tested using a gait simulator which imitates the human gait cycle, using weights of 50, 70 and 90kg. | - | It was shown that internal pressure within the socket would recover to the initial state through the active control system. | n/a |
| Inflatable Bladders / Variable Stiffness | [Ogawa, Obinata (62)](#_ENREF_62) | Custom | TT | Conformable | Automatic | Volume fluctuation | Design: Consists of several fluid filled bladders.  Control: Fluid is constantly circulated in and out of the bladder to maintain constant pressure. Fluid characteristics can be varied by applying a magnetic field.  Safety: Not Specified | Healthy participants determine pressure limits. Users set prosthesis bladder volumes. Internal socket pressure and subjective pain recorded while ambulating. | 1 | Reduce pressure and pain levels. Performance of the socket is better than conventional sockets because it is controllable in size and viscosity. | Case Study |
| Moveable Panels - Floating | [Ballesteros, Youngblood (63)](#_ENREF_63) | Custom | TT | Rigid – Single DOF | Automatic | Fluid retention | Design: 3 panels situated at the anterior medial, anterior lateral, and posterior midline of a conventional moulded socket. Control: The position of the panels is controlled relative to the point in the gait cycle using motors which are placed within an external attached housing.  Safety: Not Specified | The panels were set to pull outwards cyclically during gait to minimise fluid volume loss (outwards during late stance and returned during swing). The participants conducted 3 cycles of sitting (5mins) and walking (8mins) | 12 | 8/12 participants showed greater residual limb fluid retention. | Repeated Measures |
|  | [Larsen, McLean (64)](#_ENREF_64) |  |  |  |  |  |  | Cycles of sitting and walking bouts where the panels were released during the rest periods. Bioimpedance used to measure limb fluid volume. | 12 | Panel-pull was effective in increasing limb fluid volume. | Repeated Measures |
|  | [Weathersby, Vamos (65)](#_ENREF_65) |  |  |  |  |  |  | Cycles of sitting and walking bouts where the panels and locking pin were released during the rest periods. Bioimpedance used to measure limb fluid volume. | 6 | Auto-adjusting socket maintained good fluid stability. | Repeated Measures |
|  | [Coburn, DeGrasse (66)](#_ENREF_66) |  |  |  |  |  |  | Used magnets and iron filings in liner to attach panel; investigated how far the panel could be pulled with magnets still attached. | 4 | Magnetic method worked effectively, design need reducing in size to enable more longitudinal testing | Case Study |
| Moveable Panels – Floating & Adjustable length | [Baumgartner (67)](#_ENREF_67) | Off-the-Shelf | TR | Conformable | Manual | Volume fluctuation - Prosthesis for intervening time during rehab | Design: Rigid external frame, with rails to facilitate adjustable length, has internal soft padding and 2 adjustable soft panels located on the forearm and bicep locations. Control: the adjustable panels are controlled by micro-adjustment dials; adjustable length is through manual adjustment of screws. Safety: Not specified. | Amputees and clinicians tried the concept device and gave questionnaire feedback on their thoughts of usefulness; design; ease of use etc. | 13 (4 amputees) | Participants thought a prosthesis of this type would be useful within clinics and potentially for children. They also provided feedback on the design, helping produce the 2nd generation concept.  Adjustment dials criticised for protruding too far. | Case Study |
| Moveable Panels - Floating | [Brownsey and Fillauer (68)](#_ENREF_68) | Custom | TF | Rigid – Multi-DOF | Manual | Volume fluctuation | Design Flexible panel on the anterolateral aspect of the socket.  Control: Velcro straps.  Safety: Not Specified | 21 participants supplied with this socket for everyday use. | 21 | 11/21 now use a conventional prosthesis, 8/21 still used the adjustable socket with 6 planning to use a conventional prosthesis in the future. Devices successfully aided with early ambulation. | Cohort Study |
| Moveable Panels - Floating | [Larsen, McLean (69)](#_ENREF_69) | Custom | TT | Rigid – Multi-DOF | Manual, controlled using an app | Fluid retention | Design: 3 panels situated at the anterior medial, anterior lateral, and posterior midline of a conventional moulded socket.  Control: Panels are controlled by motors positioned between prosthetic shank and socket components. Panels position is controlled by the participant using an app.  Safety: Not Specified | Participants who could walk for at least 20 minutes without rest. Outdoor walking trials on asphalt. | 12 | Incremental socket volume increases during activity was found to be effective in increasing limb fluid volume. | Case Study |
|  | [McLean, Redd (70)](#_ENREF_70) |  |  |  |  |  |  | Socket size and residual limb fluid volume were recorded whilst participants walked on a treadmill. Adjustments were made to the panels on the socket using the app to find the optimum fit. | 10 | Small continual adjustments in socket size may be used to help maintain fluid levels if they avoid fit issues. | Repeated Measures |
|  | [McLean, Larsen (71)](#_ENREF_71) |  |  |  |  |  |  | Participants who could walk for at least 20 minutes. Indoor sitting and walking trials on a treadmill. Adjustments to panels were made to help fit and a locking pin was used to provide further adjustment. | 12 | Augmenting panel release with pin release may be an effective accommodation strategy for prosthesis users with transtibial amputation to better retain limb fluid volume. | Repeated Measures |
|  | [Weathersby, Garbini (72)](#_ENREF_72) |  |  |  | Automatic |  | Design: 3 panels situated at the anterior medial, anterior lateral, and posterior midline of a conventional moulded socket.  Control: Motors positioned between prosthetic shank and socket components adjust the panel position. Automatic control system was implemented to adjust the socket size based on the distance between the limb and socket.  Safety: Not Specified | Walk on a treadmill to characterise the system’s ability to maintain a good fit. | 10 | The socket fit was maintained by the control system and future work will involve testing outside of a controlled, laboratory environment. | Case Study |
| Moveable  Panels - Floating | [Wilson, Schuch (112)](#_ENREF_112) | Custom | TT | Rigid – Multi-DOF | Manual | Oedema Volume Fluctuations | Design: large posterior panel  Control: two lateral hose clamps (straps), manual  Safety: Not Specified | 5 participants, 1–3-year amputation maturity with volume fluctuations. | 6 | Comfort wasn’t compromised by the adjustable socket section, proved useful in managing volume fluctuating patients | Cohort Study |
| Moveable  Panels - Floating | [Schofield, Schoepp (73)](#_ENREF_73) | Custom – RevoFit  ([45](#_ENREF_45)) | TH | Rigid –Multi-DOF | Manual | Volume fluctuation & electrode contact | Design: Rigid, moulded socket with a panel at the posterior upper arm.  Control: This panel is adjusted using cabling and a RevoFit tensioning dial. The dial could be unlocked to relieve pressure during donning and doffing as well as being adjusted during use to help with fit and residual limb volume changes.  Safety: Not Specified | Uses myoelectric control, in everyday life for 11 months prior to publication. | 1 | No change in heaviness, temperature and don/doffing time compared to previous socket. Improved myoelectric control and increased prosthetic usage and satisfaction but the participant didn’t typically adjust the tightness of the socket during use. | Case Study |
| Moveable  Panels - Hinged | [Brzostowski, Larsen (74)](#_ENREF_74) | Custom – RevoFit  ([45](#_ENREF_45)) | TT | Rigid – Single DOF | Manual | Fluid retention | Design: RevoFit posterior panel  Control: Position is controlled by caballing within the socket and a posterior dial  Safety: Not Specified | Cycles of sitting and 2-minute walking within a laboratory whilst the fluid volume of the residual limb was monitored. | 15 | Releasing the socket during resting after activity reduced subsequent residual limb fluid loss. Returning the socket volume to a slightly larger size after socket and pin release reduced long-term fluid loss. | Repeated Measures |
| Moveable  Panels - Hinged | [Pinzur, Angelico (75)](#_ENREF_75) | Custom | AD | Rigid – Single DOF | Manual | Volume Fluctuation and shape change | Design: larger posterior panel hinged at proximal end of the socket  Control: Manual changes of two Velcro straps  Safety: Not specified | n/a | - | n/a | n/a |
| Moveable  Panels - Hinged | [Lanahan, Coburn (116)](#_ENREF_116) | Custom | TT | Rigid – Single DOF | Manual | Fluid retention | Design: large posterior panel hinged at distal end of the socket  Control: Micro adjustment dial used to control panel location  Safety: Only released when seated and tightened to cast socket shape. | Adjustable panel and pinlock suspension released during rest to investigate fluid retention, measured using bioimpedance. | 13 | 4min release period induces fluid recovery in 77% of participants. | Repeated Measures |
| Moveable Panels – Floating, and Circumferential Adjustment - Struts | [Huang, Wang (76)](#_ENREF_76), [Wang, Nong (77)](#_ENREF_77) | Modular | Theoretical | Rigid – Single DOF | Panels – Auto, Circumferential - Manual | Volume fluctuation, heat dissipation and constant contact force | Design: Shape Memory Alloy used to provide constant force on adjustable panels located in 4 places around the socket; adjustable straps used to vary socket circumference at fitting. Control: CA is manually by prosthetist at fitting, MP is automatic. Safety: Shape memory allow component has to be designed to suit the volume/radius change expected to be seen for each participant. | FEA study on various limb sizes to check force applied. | - | - | n/a |
| Circumferential Adjustment – Gap/Overlap | [Dillingham, Kenia (78)](#_ENREF_78) | iFIT Socket ([38](#_ENREF_38)) – Off-the-shelf | TT | Rigid- Single DOF | Manual | Volume fluctuation & shape changes | Design: Custom liner with circumferential adjustment to the socket.  Control: 2/3 posterior straps to adjust socket circumference  Safety: Not Specified | Gait and pressures within the socket were assessed in a laboratory. | 22 | No significant difference in gait between adjustable and conventional socket. Peak pressure values were lower in the adjustable socket | Cohort Study |
|  | [Kenia, Wolf (79)](#_ENREF_79) |  |  |  |  | Low resource context. Quicker and easier fitting. Less clinical demand. |  | Low resource context. Trained and fitted with device and then used in their everyday life before a follow up 7 months to 2 years later and interviewed | 6 | 5/6 still used their socket for daily use. No component failures despite use in rugged environments. The novel care delivery model was deemed a success. | Cohort Study |
|  | [McCloskey, Kenia (80)](#_ENREF_80) |  |  |  |  | Self-reported outcome measure improvement |  | Adapted version of the PEQ used, filled out at fitting and after 2 weeks of use. | 27 | iFIT socket compared to their own showed better self-reported comfort and satisfaction results. | Cohort Study |
| Circumferential Adjustment (Struts) and  Socket Length | [Hallworth, Austin (81)](#_ENREF_81) | Custom | TH | Rigid – Single DOF | Manual | Comfort & functionality | Design: Interior cushions are arranged circumferentially around the socket.  Control: Straps around the socket can be adjusted to adjust the diameter. The length of the socket can also be adjusted by clicking them into different positions using locating pins.  Safety: Not Specified | Laboratory-based testing which involved range of motion, function, mechanical performance and myoelectric control-based assessments. | 2 | Comparable fit and comfort with a conventional socket but more durable and pain free due to less abrasion and irritation. Passed static loading requirements and was similarly functional to a conventional suction socket. Socket was reported as being more mentally demanding to use. | Case study |
| Circumferential Adjustment – Gap/Overlap | [Irons, Mooney (82)](#_ENREF_82) | Custom | TF | Rigid – Single DOF | Manual | Volume fluctuation & proximal tissue load tolerance | Design: Socket is made up of two halves which can be tightened or loosened together.  Control: Velcro straps determine the tightness of the socket  Safety: Not Specified | Supplied with this socket for everyday use. | 44 | Adjustability was of benefit to enabling those who would otherwise be unable to walk, to walk. The majority of participants were able to walk further than previously. | Cohort Study |
| Circumferential Adjustment – Gap/Overlap | [Kawamura, Hayashi (83)](#_ENREF_83) | Custom | TF | Rigid – Single DOF | Manual | Large volume fluctuations due to chemotherapy | Design: 2 slits within the prosthetic socket which can be adjusted in size (pulled together).  Control: Slits are adjusted by adjusting screws/clamps which can only be implemented by a physio or prosthetist.  Safety: Not Specified: | Supplied with this socket for everyday use. Physio would adjust socket size for participants based on their limb volume. | 16 | Not suitable for immediate fitting post-surgery for individuals receiving chemotherapy. Use of the socket enabled walking with one crutch and improved psychological state. | Cohort Study |
| Circumferential Adjustment - Struts | [Kahle, Klenow (84)](#_ENREF_84) | Infinite Socket TF ([41](#_ENREF_41))  TF – Modular, TT Custom | TF | Rigid – Single DOF | Manual | Volume fluctuation & improved functionality | Design: Socket consists of 4 individual struts  Control: Struts are constrained by a tensioner which can be adjusted to control the socket diameter.  Safety: Not Specified | Performance tests: L-test, 4 square step test, 2 minute walk test. Subjective tests/ questionnaires: Socket comfort score, pain scale. | 1 | Adjustable socket was better than a conventional socket from both performance and subjective aspects. | Case Study |
|  | [Mitton, Kulkarni (85)](#_ENREF_85) |  |  |  |  |  |  | Supplied with this socket for everyday use. Results assessed using a variety of questionnaires and metrics. | 1 | SIGAM mobility score rose from C-E, socket comfort score increased from 4-8. Participant surpassed rehab goals wore prosthesis 8+ hours daily including walking indoors and outdoors. Improved function and comfort. | Case Study |
| Struts (TF) & Floating Panel (TT) | [Isaacson, Lin (86)](#_ENREF_86) |  | TT & TF | Single (TF) and Multi (TT) DOF |  |  |  | At the time of socket fitting, patients were fit with a standard thermoplastic socket and the Infinite Fit socket, standard functional outcome measures compared | 127 | Improved comfort scores, may allow for improved functional outcomes and mobility. | Cohort Study |
| Circumferential Adjustment (Gap/Overlap) and Socket Length | [Nia, Toetschinger (87)](#_ENREF_87) | Varos Socket ([43](#_ENREF_43)) – Off-the-shelf | TF | Rigid – Single DOF | Manual | Volume fluctuation | Design: 2 shells which can move relative to each other.  Control: Cables adjust the fit of the socket and can be set by the prosthetist at 2 discrete levels which the user can switch between. Length can also be adjusted when the socket is not being worn.  Safety: Not Specified | Rehabilitation intervention study between standard socket and PFSV socket. Socket fit, pain comfort and satisfaction compared. | 10 | Increased stability, suspension, comfort and socket fit. Increased user satisfaction and a reduction in socket related pain in the residual limb was reported by participants when compared to their conventional prosthesis. | Cast Study |
| Circumferential Adjustment (Gap/Overlap) and Socket Length | [Sathishkumar, Manigandan (88)](#_ENREF_88) | Custom | TF | Rigid – Single DOF | Manual | Promote ambulation ASAP after surgery & reduce cost | Design: Support on all 4 sides.  Control: Adjust length, lateral sliding girth adjuster which allows for various residual limb diameter, and leather/Velcro conformer which tightens and loosens the socket (diameter) appropriately.  Safety: Not Specified | n/a | - | n/a | n/a |
| Circumferential Adjustment – Gap/Overlap | [Takakura, Akasaka (89)](#_ENREF_89) | Custom | TF | Conformable | Manual | Volume fluctuation | Design:  Control: Circumferential straps which can adjust the diameter of the socket by +/-3cm.  Safety: Not Specified | Participants with unstable residual limb volumes were supplied with this socket for everyday use. | 4 | No pain or looseness experienced from the socket. 2 participants needed assistive devices and 2 could walk without any assistance. | Case Study |
| Circumferential Adjustment – Gap/Overlap | [Tingleff and Jensen (90)](#_ENREF_90) | Custom | KD | Rigid – Single DOF | Manual | Volume fluctuation & accommodate bulbous distal end | Design: Carbon fibre socket with Kevlar fibres within resin.  Control: A buckle system which can adjust the circumference of the socket.  Safety: Not Specified | Case study of a single participant 9 years post amputation. Used socket in everyday life but also for athletic activities. | 1 | Improved prosthetic suspension and athletic performance. | Case Study |
| Circumferential Adjustment - Struts | [Terrazas Quezada (91)](#_ENREF_91) | Custom | TT | Rigid – Single DOF | Manual | Volume fluctuation | Design: Distal end of the socket consists of 4 panels which are fitted to a disc.  Control: Disc can be manually altered by the prosthetist to adjust the circumference of the distal end of the socket. Straps are positioned along the length of the socket and can be adjusted by the user.  Safety: Not Specified | Pressure values within the socket recorded during several periods of ambulation | 1 | Socket comfort was good but there were issues regarding the panels moving during ambulation. | Case Study |
|  | [Vaughan (92)](#_ENREF_92) |  |  |  |  |  |  | n/a | - | n/a | n/a |
|  | [Vaughan (93)](#_ENREF_93) |  |  |  |  |  |  | n/a | - | n/a | n/a |

*(Locations: TT-Trans-tibial, TF-Trans-femoral, TR-Trans-radial, TH-Trans-humeral, KD-Knee Disarticulation, AD-Ankle Disarticulation)*

# References

1. Cornell, KD. *Adjustable Prosthetic Limb Socket.* US patent 2012041567A1. 2012.

2. Mahon, JA. *Adjustable Prosthetic Interfaces and Related Systems and Methods.* US patent 2011071647A1. 2011.

3. Ballas, GJ, Ballas, MT. *Modular Adjustable Prosthetic Volume Compensation System.* US patent 2022/0110768 A1. 2022.

4. Ballas, GJ, Ballas, MT. *Adaptive Compression Prosthetic Socket System and Method.* US patent 10357382B2. 2019.

5. Brown SR, RN. *Adjustable post-operative preparatory prosthetic system.* US patent 5728165A. 1998.

6. Dillingham, TR. *Modular Prosthesis System.* US patent 2012259433A1. 2012.

7. Dillingham, TR. *Above-The-Knee Modular Prosthesis System.* WO patent 2013056203A2. 2013.

8. Dillingham, TR. *Modular prosthetic devices and prosthesis systems.* US patent 11382775B2. 2022.

9. Cespedes, JJ, Hurley, GR, Williams, JR. *Prosthetic socket with an adjustable height ischial seat.* US patent 2016058584A1. 2016.

10. Martin, JJ. *Air Hammock for a Prosthetic.* US patent 2020397602A1. 2020.

11. Bache, A. *Adjustable Socket System.* US patent 2012101597A1. 2012.

12. Bache, A, Tuttle, MP. *Adjustable prosthetic limb system.* US patent 10543112B2. 2020.

13. Will, C, Finke, LB. *Prosthesis socket and method for controlling an adjustment of an inner circumference of a prosthesis socket.* US patent 2019183663A1. 2019.

14. Kuniholm, J, Meyer, Z. *Variable compliance prosthetic socket with breathable matrix.* US patent 2021298928A1. 2021.

15. Sanders, JE, Garbini, JL, McLean, J, Hinrichs, P, Brzostowski, J, Redd, CB, Cagle, J, Bennett, S, Wang, H. *Motorized adjustable socket for amputee prosthesis users and methods for use thereof.* US patent 2020345520A1. 2020.

16. Smith, A, Hogan, P, Labelle, J. *Volume Adjustable Transtibial Socket.* US patent 11173057B2. 2021.

17. Kaluf, BD, Williams, J, Karns, SL, Freed, RD, Russell, MA. *Prosthetic Limb Having an Adjustable Socket.* WO patent 2015013560A1. 2015.

18. Wang, L, Zhang, C, Li, D, Tian, X, Sun, C, Tang, L. *3D printing flexible socket with adaptability.* CN patent 108451676A. 2018.

19. Marks, ECR. *Improvements in Artificial Limbs.* GB patent 109085A. 1917.

20. Cottingham, HV, Scrocco, J. *Adjustable prosthetic limb.* US patent 4161042A. 1979.

21. Laszczak, P, Moser, D, Gallego, MAI, McCarthy, JR, Zahedi, MS. *Prosthetic Limb Socket.* US patent 2021113356A1. 2021.

22. Thompson, H, Shorter, JJ. *Artificial Limb Socket.* GB patent 2103490A. 1983.

23. Livni, A, Wolfus, S, Wolfus, O. *Dynamic Adjustable Socket for Prosthetics.* WO patent 2015103506A1. 2015.

24. Prescott, DR, Sullivan, LK, Fleming, AJ. *Modular, Adjustable, Above-Knee Prosthetic Socket.* US patent 2020297514A1. 2020.

25. Marx, HW, Marx, N. *Fluctuating volume adjustable preparatory below knee prosthetic socket.* US patent 4842608A. 1989.

26. Fӧrster, H, Heublein, C. *Prosthesis Assembly.* US patent 2014243996A1 2014.

27. Gair JR, JL. *Modular Prosthesis System.* US patent 2021353441A1. 2021.

28. Dean, RC, Mayor, MB, Nelson, DF, Braley, CS, Blanchford, MW. *Dynamic variable geometry fitting system for use with a body appliance.* US patent 2002099450A1. 2002.

29. Rajewski, B. *An Adjustable Prosthetic Socket and Suspension System.* WO patent 2020078864A1. 2020.

30. Galea, AM, Leroy, K, Truong, TQ. *Active Prosthetic Socket.* US patent 2012271433A1. 2016.

31. Epoch Medical Innovations Inc. Limb Air Product Overview [Internet]. 2022 [cited 13/03/2023]. Available from: <https://www.epochmedical.com/copy-of-limbair>.

32. Quorum Prosthetics. The Quatro [Internet]. 2019 [cited 07/12/22]. Available from: <https://opquorum.com/programs/>.

33. LIM Innovations. Infinite Socket TT-S [Internet]. 2022 [cited 07/12/22]. Available from: <http://www.liminnovations.com/infinite-socket-tts/>.

34. Martin Bionics. Martin Bionics Prosthetics + Research [Internet]. 2022 [cited 07/12/22]. Available from: <https://martinbionics.com/>.

35. Stumpworx. Stumpworx [Internet]. 2022 [cited 07/12/22]. Available from: <https://www.stumpworx.com/>.

36. CJ Socket Technologies. CJ Socket Technologies - The CJ Socket Solution [Internet]. 2022 [cited 07/12/22]. Available from: <https://cjsocket.com/>.

37. Flo Tech. Flo-Tech-Tor [Internet]. 2022 [cited 07/12/22]. Available from: <https://1800flo-tech.com/products/trans-tibial-products/flo-tech-tor/>.

38. iFit Prosthetics. iFIT Transtibial System [Internet]. 2022 [cited 07/12/22]. Available from: <https://www.ifitprosthetics.com/transtibial-socket.html>.

39. KOALAA. The ALX [Internet]. 2022 [cited 07/12/22]. Available from: <https://www.yourkoalaa.com/the-alx>.

40. Ossur. Connect TF [Internet]. 2022 [cited 07/12/22]. Available from: <https://www.ossur.com/en-gb/prosthetics/sockets/connect-tf>.

41. LIM Innovations. Infinite Socket TF [Internet]. 2022 [cited 07/12/22]. Available from: <http://www.liminnovations.com/infinite-socket-tf/>.

42. Delft Prosthetics. Wilmer Open Fitting [Internet]. 2022 [cited 07/12/22]. Available from: <https://www.delftprosthetics.nl/en/open-socket/product-info-open-socket>.

43. Ottobock. Varos Socket [Internet]. 2022 [cited 07/12/22]. Available from: <https://shop.ottobock.us/Prosthetics/Lower-Limb-Prosthetics/Socket-Technologies-Liners/Varos-Socket/Varos/p/5A60>.

44. Toughware PRX. International Transradial Adjustable Limb (ITAL) [Internet]. 2022 [cited 07/12/22]. Available from: <https://toughwareprx.com/product/international-transradial-adjustable-limb-ital/>.

45. Click Medical. Empowered Amputees are More Comfortable, More Mobile, and Happier [Internet]. 2022 [cited 07/12/22]. Available from: <https://clickmedical.co/amputees/>.

46. Candrea D, Sharma A, Osborn L, Gu YK, Thakor N, Ieee, editors. An Adaptable Prosthetic Socket: Regulating Independent Air Bladders Through Closed-Loop Control. IEEE International Symposium on Circuits and Systems (ISCAS); 2017 May 28-31; Baltimore, MD. NEW YORK: Ieee; 2017.

47. Carrigan W, Nothnagle C, Savant P, Gao F, Wijesundara MBJ, Ieee, editors. Pneumatic Actuator Inserts for Interface Pressure Mapping and Fit Improvement in Lower Extremity Prosthetics. 6th IEEE International Conference on Biomedical Robotics and Biomechatronics (BioRob); 2016 Jun 26-29; Singapore. NEW YORK: Ieee; 2016.

48. Greenwald RM, Dean RC, Board WJ. Volume management: smart variable geometry socket (SVGS) technology for lower-limb prostheses. Journal of Prosthetics & Orthotics (JPO). 2003;15(3):107-12.

49. Gu Y, Yang D, Osborn L, Candrea D, Liu H, Thakor N. An adaptive socket with auto-adjusting air bladders for interfacing transhumeral prosthesis: A pilot study. Proceedings of the Institution of Mechanical Engineers Part H, Journal of engineering in medicine. 2019;233(8):812-22.

50. Ibrahimi M, Gruppioni E, Menciassi A. Variable stiffness and shape prosthetic socket based on layer jamming technology. Piscataway: The Institute of Electrical and Electronics Engineers, Inc. (IEEE); 2022.

51. Ma PH, Foca FJ, Powermininni M, Sumell A. AN ADJUSTABLE VOLUME SOCKET BELOW KNEE PROSTHESIS FOR GERIATRIC-PATIENTS - A PRELIMINARY-REPORT. Arch Phys Med Rehabil. 1987;68(9):594-.

52. Mandić V, Manev S, Cvitanović V, Zovko I, Horcicka M. [Prosthesis for tibial amputation with adaptable socket]. Revue de chirurgie orthopedique et reparatrice de l'appareil moteur. 1965;51(7):645-7.

53. Mbithi FM, Chipperfield AJ, Steer JW, Dickinson AS. Developing a control framework for self-adjusting prosthetic sockets incorporating tissue injury risk estimation and generalized predictive control. Biomedical Engineering Letters. 2022;12(1):59-73.

54. Mbithi FM. Developing a Framework for an Adaptive Transtibial Prosthetic Socket Using Fea-Based Tissue Injury Risk Estimation and Generalised Predictive Control [Ph.D.]. Ann Arbor: University of Southampton (United Kingdom); 2020.

55. Montgomery JT, Vaughan MR, Crawford RH. Design of an actively actuated prosthetic socket. Rapid Prototyping J. 2010;16(3):194-201.

56. Razak NA, Osman NA, Gholizadeh H, Ali S. Prosthetics socket that incorporates an air splint system focusing on dynamic interface pressure. BioMedical Engineering OnLine. 2014;13(1):108.

57. Abd Razak N, Osman NA, Ali S, Gholizadeh H, Abas WW. Comparison study of the prosthetics interface pressure profile of air splint socket and ICRC polypropylene socket for upper limb prosthetics. Biocybernetics and Biomedical Engineering. 2015;35(2):100-5.

58. Sanders JEP, Cagle JCBSE, Harrison DSBS, Myers TRMME, Allyn KJCPO. How does adding and removing liquid from socket bladders affect residual-limb fluid volume? J Rehabil Res Dev. 2013;50(6):845-60.

59. Sang YJ, Li X, Luo Y. Characteristics of a volume-adjustable compression chamber for transradial prosthetic interface. Proc Inst Mech Eng Part H-J Eng Med. 2016;230(7):650-60.

60. Sang YJ, Li X, Gan Y, Su D, Luo Y. A novel socket design for upper-limb prosthesis. Int J Appl Electromagn Mech. 2014;45(1-4):881-6.

61. Seo JH, Lee HJ, Seo DW, Lee DK, Kwon OW, Kwak MK, et al. A Prosthetic Socket with Active Volume Compensation for Amputated Lower Limb. Sensors. 2021;21(2):17.

62. Ogawa A, Obinata G, Hase K, Dutta A, Nakagawa M, Ieee, editors. Design of Lower Limb Prosthesis with Contact Pressure Adjustment by MR Fluid. 30th Annual International Conference of the IEEE-Engineering-in-Medicine-and-Biology-Society; 2008 Aug 20-24; Vancouver, CANADA. NEW YORK: Ieee; 2008.

63. Ballesteros D, Youngblood RT, Vamos AC, Garbini JL, Allyn KJ, Hafner BJ, et al. Cyclic socket enlargement and reduction during walking to minimize limb fluid volume loss in transtibial prosthesis users. Medical Engineering & Physics. 2022;103:9.

64. Larsen BG, McLean JB, Brzostowski JT, Carter R, Allyn KJ, Hafner BJ, et al. Does actively enlarging socket volume during resting facilitate residual limb fluid volume recovery in trans-tibial prosthesis users? Clinical Biomechanics. 2020;78:7.

65. Weathersby EJ, Vamos AC, Larsen BG, McLean JB, Carter RV, Allyn KJ, et al. Performance of an auto-adjusting prosthetic socket during walking with intermittent socket release. J Rehabil Assist Technol Eng. 2022;9:14.

66. Coburn KA, DeGrasse NS, Allyn KJ, Larsen BG, Garbini JL, Sanders JE. Using magnetic panels to enlarge a transtibial prosthetic socket. Medical engineering & physics. 2022;110:103924.

67. Baumgartner BL. Design of an adjustable temporary socket for a transradial prosthesis: Wien; 2022.

68. Brownsey WS, Fillauer C. Temporary prosthesis with adjustable socket. Physical therapy. 1967;47(12):1129-31.

69. Larsen BG, McLean JB, Allyn KJ, Brzostowski JT, Garbini JL, Sanders JE. How do transtibial residual limbs adjust to intermittent incremental socket volume changes? Prosthet Orthot Int. 2019;43(5):528-39.

70. McLean JB, Redd CB, Larsen BG, Garbini JL, Brzostowski JT, Hafner BJ, et al. Socket size adjustments in people with transtibial amputation: Effects on residual limb fluid volume and limb-socket distance. Clinical Biomechechanics. 2019;63:161-71.

71. McLean JB, Larsen BG, Weathersby EJ, Carter RV, Allyn KJ, Garbini JL, et al. Fluid Volume Management in Prosthesis Users: Augmenting Panel Release with Pin Release. Pm&R. 2020;12(12):1236-43.

72. Weathersby EJ, Garbini JL, Larsen BG, McLean JB, Vamos AC, Sanders JE. Automatic Control of Prosthetic Socket Size for People With Transtibial Amputation: Implementation and Evaluation. IEEE Trans Biomed Eng. 2021;68(1):36-46.

73. Schofield JS, Schoepp KR, Stobbe M, Marasco PD, Hebert JS. Fabrication and application of an adjustable myoelectric transhumeral prosthetic socket. Prosthetics & Orthotics International. 2019;43(5):564-7.

74. Brzostowski JT, Larsen BG, Youngblood RT, Ciol MA, Hafner BJ, Gurrey CJ, et al. Adjustable sockets may improve residual limb fluid volume retention in transtibial prosthesis users. Prosthetics & Orthotics International. 2019;43(3):250-6.

75. Pinzur MS, Angelico JA, Quigley MJ. A volume-adaptable prosthesis for ankle disarticulation. JPO: Journal of Prosthetics and Orthotics. 1993;5(3):77.

76. Huang Y, Wang M, Yu H. The design of the adaptive prosthetic socket. Int J Appl Electromagn Mech. 2023;71(S1):S561-S9.

77. Wang M, Nong Q, Qian Y, Huang Y, Wang Y, Yu H. Design of Adjustable Frame-Type Prosthetic Socket for Lower Limb. IRBM. 2023;44(1):100731.

78. Dillingham T, Kenia J, Shofer F, Marschalek J. A Prospective Assessment of an Adjustable, Immediate Fit, Transtibial Prosthesis. PM & R : the journal of injury, function, and rehabilitation. 2019;11(11):1210-7.

79. Kenia J, Wolf B, Marschalek J, Dillingham T. An Immediate Fit, Adjustable, Modular Prosthetic System for Addressing World-Wide Limb Loss Disability. Archives of rehabilitation research and clinical translation. 2021;3(2):100120.

80. McCloskey C, Kenia J, Shofer F, Marschalek J, Dillingham TR. Improved Self-Reported Comfort, Stability, and Limb Temperature Regulation With an Immediate Fit, Adjustable Transtibial Prosthesis. Archives of rehabilitation research and clinical translation. 2020;2(4):100090.

81. Hallworth BW, Austin JA, Williams HE, Rehani M, Shehata AW, Hebert JS. A Modular Adjustable Transhumeral Prosthetic Socket for Evaluating Myoelectric Control. IEEE J Transl Eng Health Med-JTEHM. 2020;8:0700210.

82. Irons G, Mooney V, Putnam S, Quigley M. LIGHTWEIGHT ABOVE-KNEE PROSTHESIS WITH AN ADJUSTABLE SOCKET. Orthotics and Prosthetics. 1977;31(1):3-15.

83. Kawamura J, Hayashi Y, Yoneda T, Minamihara K, Tanaka H, Arimitsu K, et al. Temporary above-knee prostheses and training programme during chemotherapy. Prosthetics and orthotics international. 1985;9(2):87-91.

84. Kahle JT, Klenow TD, Highsmith MJ. COMPARATIVE EFFECTIVENESS OF AN ADJUSTABLE TRANSFEMORAL PROSTHETIC INTLERFACE ACCOMMODATING VOLUME FLUCTUATION: CASE STUDY. Technol Innov. 2016;18(2/3):175-83.

85. Mitton K, Kulkarni J, Dunn KW, Ung AH. Fluctuating residual limb volume accommodated with an adjustable, modular socket design: A novel case report. Prosthetics and orthotics international. 2017;41(5):527-31.

86. Isaacson BM, Lin SC, Rothberg DL. Improvement in functional outcomes with the infinite socket. JPO: Journal of Prosthetics and Orthotics. 2018;30(4):181-6.

87. Nia A, Toetschinger G, Kubinec T, Domayer S. Evaluation of the new, patient-adjustable socket system Varos in the early phase of prosthetic rehabilitation: a pilot study. Eur J Phys Rehabil Med. 2022.

88. Sathishkumar S, Manigandan C, Asha T, Charles J, Poonoose RP. A cost-effective, adjustable, femoral socket, temporary prosthesis for immediate rehabilitation of above-knee amputation. Int J Rehabil Res. 2004;27(1):71-4.

89. Takakura Y, Akasaka K, Takahashi Y, Kikuchi E, Suyama T, Takahashi K, et al., editors. New adjustable suction socket for above-knee amputation. 1st World Congress of the International-Society-of-Physical-and-Rehabilitation-Medicine (ISPRM I); 2001 Jul 07-13; Amsterdam, Netherlands. 40128 BOLOGNA: Medimond S R L; 2001.

90. Tingleff H, Jensen L. A newly developed socket design for a knee disarticulation amputee who is an active athlete. Prosthetics and Orthotics International. 2002;26(1):72-5.

91. Terrazas Quezada S. Redesign of a Volume Adjustable Transtibial Prosthetic Socket [M.S.]. Ann Arbor: The University of Texas at El Paso; 2017.

92. Vaughan M. A low-cost volume adjustable lower limb prosthetic socket: design and evaluation (Doctoral Dissertation). Retrieved from UT Electronic Theses and Dissertations collection. 2014.

93. Vaughan MR. Design and analysis of a volume adjustable transtibial prosthetic socket for pediatric amputees in developing countries 2009.
